# Quality, topics, and demographic trends of animal systematic reviews - an umbrella review

**DOI:** 10.1186/s12967-024-05992-0

**Published:** 2025-01-06

**Authors:** Bernard Friedrich Hild, David Brüschweiler, Sophia Theodora Katharina Hild, Julia Bugajska, Viktor von Wyl, Marianna Rosso, Kimberley Elaine Wever, Eva Furrer, Benjamin Victor Ineichen

**Affiliations:** 1 Center for Reproducible Science, University of Zurich, Zurich, Switzerland; 2 Epidemiology, Biostatistics and Prevention Institute, University of Zurich, Zurich, Switzerland; 3 Institute for Implementation Science in Health Care, University of Zurich, Zurich, Switzerland; 4 Department of Anaesthesiology, Pain and Palliative Medicine, Radboud university medical center, Nijmegen, The Netherlands; 5 Department of Neuroradiology, Clinical Neuroscience Center, University Hospital Zurich, Zurich, Switzerland

**Keywords:** Translational research, Systematic review, Animal research, Neuroscience, Animal welfare, Evidence map, Automation, Risk of bias

## Abstract

**Background:**

Animal systematic reviews are critical to inform translational research. Despite their growing popularity, there is a notable lack of information on their quality, scope, and geographical distribution over time. Addressing this gap is important to maintain their effectiveness in fostering medical advancements.

**Objective:**

This study aimed to assess the quality and demographic trends of animal systematic reviews in neuroscience, including changes over time.

**Methods:**

We performed an umbrella review of animal systematic reviews, searching Medline and Embase for reviews until January 27, 2023. A data mining method was developed and validated to automatically evaluate the quality of these reviews.

**Results:**

From 18‘065 records identified, we included 1‘358 animal systematic reviews in our study. These reviews commonly focus on translational research but with notable topical gaps such as schizophrenia, other psychiatric disorders, and brain tumours. They originate from 64 countries, with the United States, China, the UK, Brazil, and Iran being the most prolific. The automated quality assessment indicated high reliability, with F1-scores over 80% for most criteria. Overall, the reviews were of high quality and the quality improved over time. However, many systematic reviews did not report a pre-registered study protocol. Reviews with a pre-registered protocol generally scored higher in quality. No significant differences in quality were observed between countries.

**Conclusion:**

Animal systematic reviews in neuroscience are of overall of high quality. Our study highlights specific areas for enhancement such as the recommended pre-publication of study protocols. It also identifies under-represented topics that could benefit from further investigation to inform translational research. Such measures can contribute to the effective translation of animal research findings to clinical applications.

**Supplementary Information:**

The online version contains supplementary material available at 10.1186/s12967-024-05992-0.

## Introduction

A systematic review is a comprehensive synthesis of existing research on a specific topic, typically including a comprehensive search, explicit in- and exclusion criteria, critical appraisal, and structured analysis of relevant studies to summarize the findings and draw evidence-based conclusions [1–3]. Originally developed for clinical research, systematic reviews are now increasingly used in preclinical fields [4, 5]. Their increasing use in this area can enhance our understanding of disease mechanisms and foster the transition from laboratory research to clinical application. This can provide insights without necessitating new animal experiments [6–9]. For example, a systematic review found that treatments tested in clinically relevant animal models (e.g., stroke drugs tested in older animals with comorbidities) are more likely to succeed in human trials, while those tested in less realistic models often fail [10]. Similarly, a pancreatitis clinical trial using probiotics showed no benefit and even higher mortality [11], likely because a systematic assessment of earlier animal studies showed different probiotics and administration regimens, highlighting discrepancies between animal models and clinical trials [12]. Systematic reviews also improve research transparency, show missing evidence, and identify needs and designs for future studies.

However, the utility of animal systematic reviews to inform human health depends on their rigour and relevance. Recent findings indicate a prevalence of overall low-quality clinical [13] but also preclinical systematic reviews [14–17]. Yet there is a lack of detailed assessment of preclinical systematic review rigour, which is increasingly challenging due to the rapid growth of biomedical literature [18]. Additionally, understanding country-specific differences in systematic review quality could guide targeted training efforts aimed at improving review standards, as advocated by international collaborations like SYRLCE and CAMARADES [19, 20]. This would not only support researcher training but also reveal trends in the most prolific countries for systematic reviews. Finally, it remains unclear to what extent preclinical systematic reviews align with topics relevant to human health, particularly prevalent diseases. Strengthening this alignment is essential for translational science to advance clinical care [21]. Further, mapping these topics could identify underexplored areas within preclinical evidence synthesis.

To address these issues, we have conducted an umbrella systematic review aimed at mapping animal systematic reviews in neuroscience, a major field within biomedical research. Our analysis aimed to answer three questions: (1) What research topics have been addressed by animal systematic reviews in neuroscience, including their translational focus? (2) Which countries are the leading producers of animal systematic reviews, and how has this changed over time? (3) What is the overall quality of these systematic reviews in animal neuroscience, does it vary across different countries, and is there an improvement in quality over time?

## Materials and methods

### Study registration

We registered a study protocol for our umbrella review [22] on the Open Science Framework platform (OSF, https://osf.io/wx5ta/) on November 25 2023. This review used the Preferred Reporting Items for Systematic Reviews and Meta-Analysis (PRISMA) guidelines for reporting [23].

### Information sources and search strategy

To identify all available animal systematic reviews in neuroscience, we searched for studies published from database inception up to January 27, 2023, on Embase and Medline (both via Ovid). The search string was created in Medline and translated to Embase (Supplementary Data). In brief, the search string was comprised of a component for systematic reviews and meta-analysis and a component for neuroscience, and was limited to animal studies by employing the SYRCLE animal filter [24].

### Eligibility criteria

#### Inclusion criteria

In line with the JBI Handbook for umbrella reviews [25], we included both systematic reviews and meta-analyses summarizing animal studies in neuroscience, as meta-analyses often accompany systematic reviews in this field [3]. These studies required at least two of the following three items to be fulfilled: (1) an explicit mention of the terms “systematic review” or “meta-analysis” in the title or abstract, or by (2) mentioning a systematic literature search in at least two databases in the abstract, and by (3) mentioning adherence to systematic review guidelines in the abstract. We selected these criteria deliberately, as authors are likely to use the terms “systematic review” or “meta-analysis” for clarity and to follow established guidelines (e.g., Cochrane, CAMARADES, SYRCLE). Additionally, a systematic review’s defining characteristic is its comprehensive literature search [3]. We also included systematic reviews synthesizing both animal and human primary data.

#### Exclusion criteria

Primary studies. Non-systematic reviews. Systematic reviews outside the neurosciences. Systematic reviews only including human, in vitro or in silico data.

### Study selection process and (automated) data extraction

Two reviewers (DB and BFH) screened titles and abstracts of studies for their relevance in the web-based application Rayyan [26]. In accordance with the study protocol, we did not review the full texts of these studies due to the anticipated high volume of eligible studies. Subsequently, we extracted the following (meta)data: title, authors, publication year, journal, and number of authors. Data on study country and author keywords were retrieved by matching DOIs of respective publications with the Embase export. Additionally, based on the abstract, systematic reviews were manually categorized based on whether they focused on translational or purely basic research questions including whether systematic reviews included only animal studies or both animal and human studies.

 We automatically extracted items related to the quality of systematic reviews from the full text. This approach was inspired by earlier suggestions [9] and further extended by additional criteria. These elements pertain either to how the systematic reviews were reported or to the quality of their methodological approach. The elements focusing on reporting include: (1) Was a study protocol drafted? (2) Was screening and/or extraction conducted by two or more reviewers? (3) Was a research question and/or study goal defined? (4) Were two or more literature databases searched? Items related to the methodological quality were: (5) Was a flowchart for study selection provided? (6) Was a conflict-of-interest statement provided? (7) Were in- and exclusion criteria reported? (8) Was a literature search date provided? (9) Was a literature search string provided? (10) Was a critical appraisal of included studies conducted? 11) Did the study mention any relevant systematic review guidelines, e.g., SYRCLE, CAMARADES, or PRISMA? For the sake of clarity, we will refer to all these items as quality items forward.

These items were automatically extracted from full texts of eligible studies using a custom-built R tool. This tool uses regular expressions, i.e., patterns of characters that define specific text matches, to match relevant keywords in the methods and results sections of the respective studies. For each of these items, we created libraries of regular expressions based on another umbrella review of 120 systematic reviews in translational biomedicine [27]. The tool segments each paper into sections (like results or methods), removes the ‘references’ section, and finally searches for matching regular expression patterns. To evaluate performance of this tool in this study, we manually examined a random 10% sample of the systematic reviews and calculated inter-rater agreement. Discrepancies were resolved by discussion. Both our regular expression libraries and the R tool are available at: https://osf.io/wx5ta/.

### Data synthesis and analysis

For the analysis of covered topics, study keywords as provided by the authors were organized in descending order of frequency and aligned with a pre-established list of neurological/psychiatric conditions. In addition, each systematic review was manually classified by two independent reviewers into a specific disease according to the disease category from the Global Burden of Disease study for neurological [28] and mental disorders [29] and assigned to one or more of the following topics: therapeutic intervention, pathophysiology and mechanisms, diagnostic tools and biomarkers, and other. To assess how well a disease is covered by preclinical systematic reviews, we calculated the ratio of systematic review counts per disease to the respective disease prevalence (multiplied by 10,000), referred to as the systematic review-disease prevalence ratio. For the analysis of quality over time and growth rate, we focused our analysis on years with > 10 systematic reviews being published per year. For the quality analysis, we assigned 1 point to each of the 11 items described above, allowing for a maximum score of 11 points and normalized the lowest and highest score to 0 and 1, respectively.

We summarized findings in narrative fashion and present descriptive statistics for demographic parameters. We conducted two statistical tests to compare quality scores, i.e., an ANOVA to compare the average systematic review quality per country and an unpaired t-test to compare the quality scores between systematic reviews with or without a pre-registered study protocol. RStudio (Version 2023.03.0, Build 386) running R Version 4.2.3 (2023-03-15 ucrt, “Shortstop Beagle”) was used for all analyses and visualizations.

## Results

### Study selection and general study characteristics

**Fig. 1:**
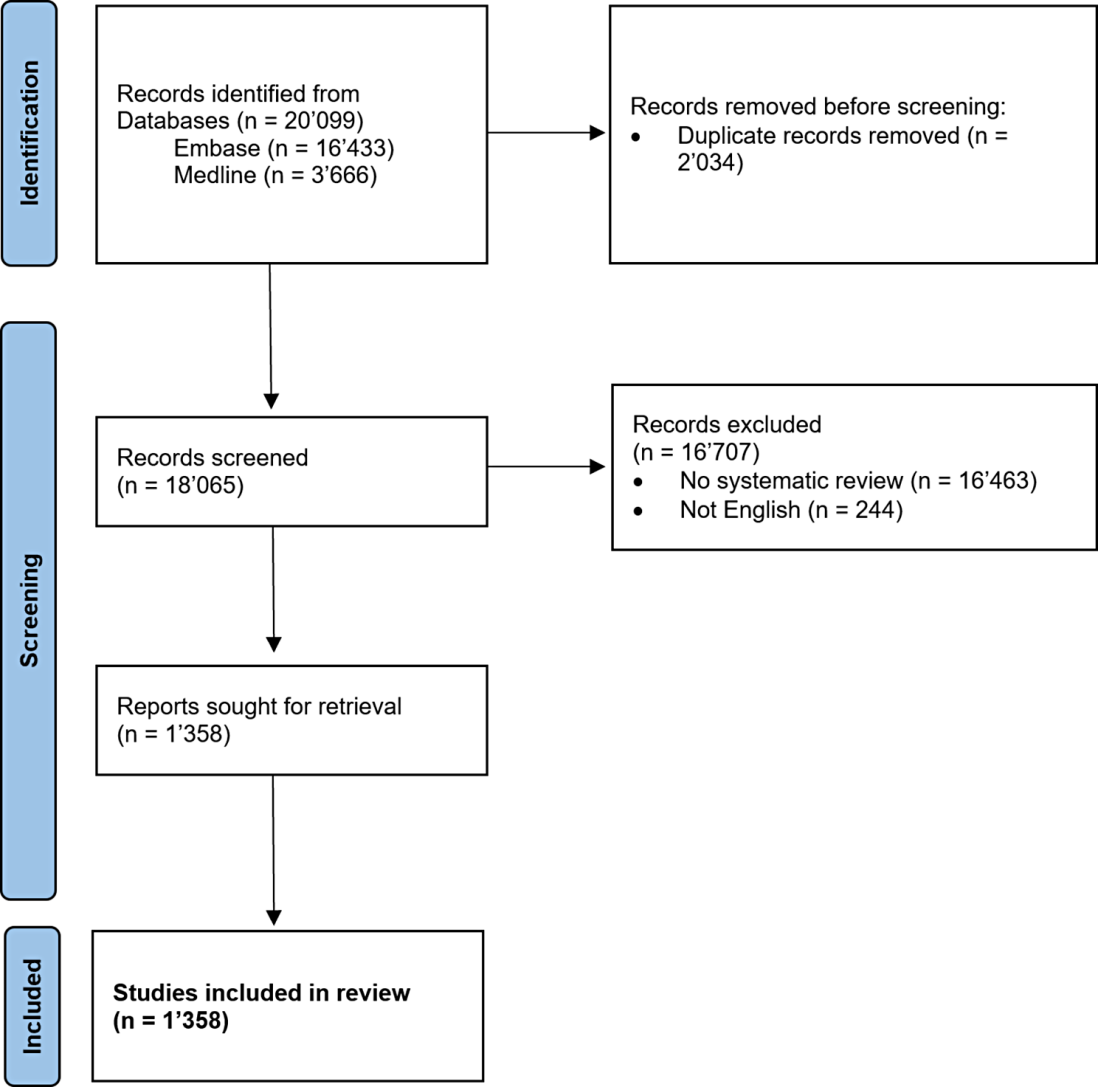
Flow chart for study inclusion

We retrieved 18’065 unique records from our database search. After title and abstract screening, we included 1’358 studies (Fig. 1), with approximately half of them containing a meta-analysis (50%). Most systematic reviews had 4 to 5 authors (median 5, range 1–64).

### Growth of the systematic review library

The first systematic review was published in 1997. Since 2007, the number of new systematic reviews in a year has grown increasingly, surmounting the number of systematic reviews published yearly from 5 in 2007 to 305 in 2022. The median global growth of systematic reviews per year was 26%.

Between 2018 and 2023, the systematic review library has more than tripled, surging from 417 systematic reviews at the start of 2018 to 1’331 SRs in 2022. Given the mean annual growth rate of 29% and assuming a steady growth, a doubling of the number of systematic reviews can be anticipated every 2.7 years (Supplementary Table 1).

### Systematic review topics

53% of the systematic reviews included in our study addressed translational research questions (*n* = 724). In contrast, relatively few systematic reviews focused on basic research (*n* = 124, 9%). Along these lines, 938 systematic reviews included only animal studies (69%) and 420 systematic reviews included both animal and human studies (31%).

**Fig. 2:**
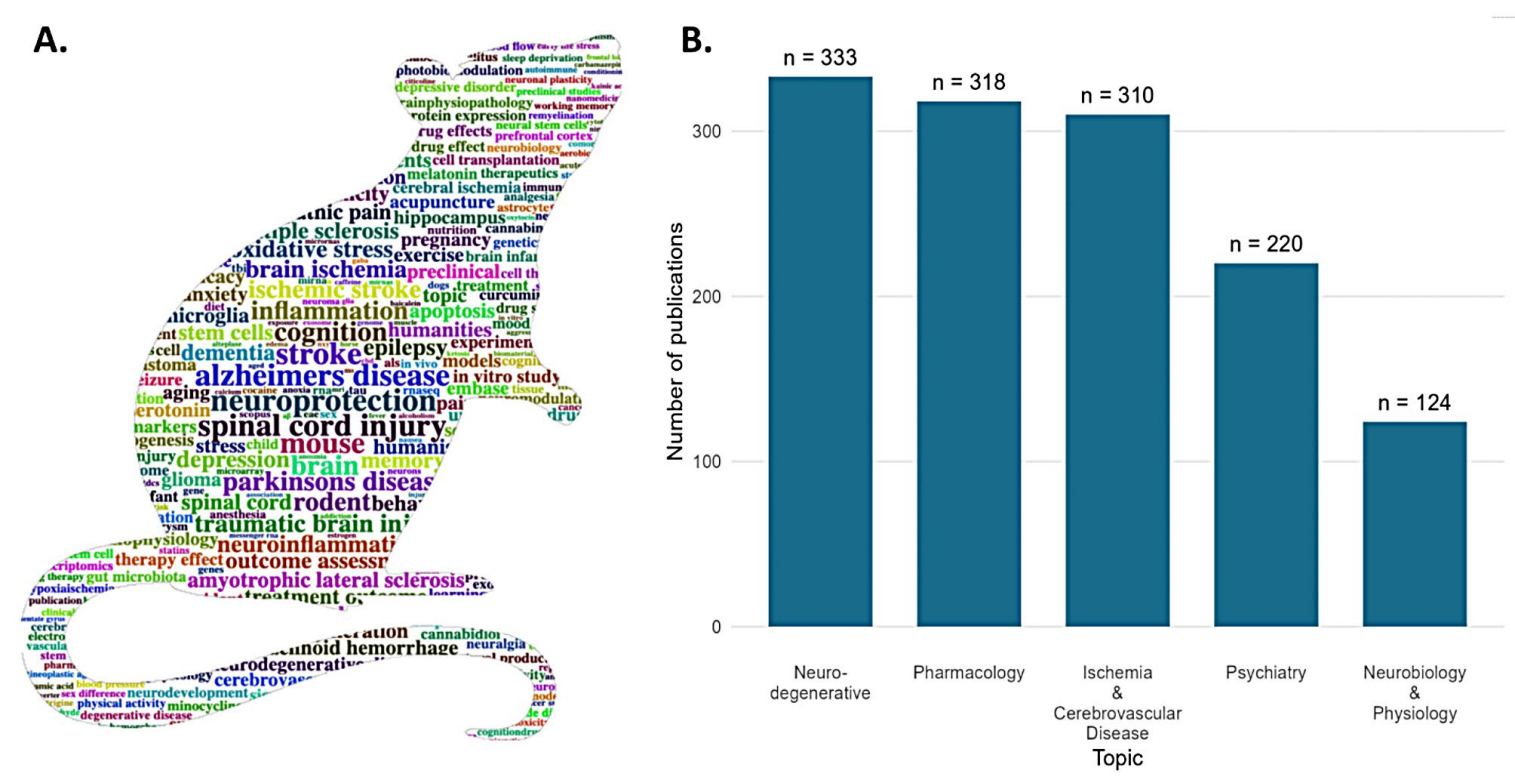
Topics addressed by animal systematic reviews. Word cloud of covered topics (**A**) and topic quantification per research domain (**B**)

The most common research areas covered were neurodegenerative diseases (*n* = 333, 25%), pharmacology (*n* = 318, 23%), ischemia/cerebrovascular disease (*n* = 310, 23%), and psychiatry (*n* = 220, 16%), constituting over two thirds of the included systematic reviews (Fig. 2). Less frequently covered areas were neurobiology and physiology (*n* = 124, 9%), neonatal and neurodevelopmental disorders (*n* = 84, 6%), neuromuscular disorders (*n* = 82, 6%), and neuro-gastroenterology (*n* = 73, 5%).

**Fig. 3:**
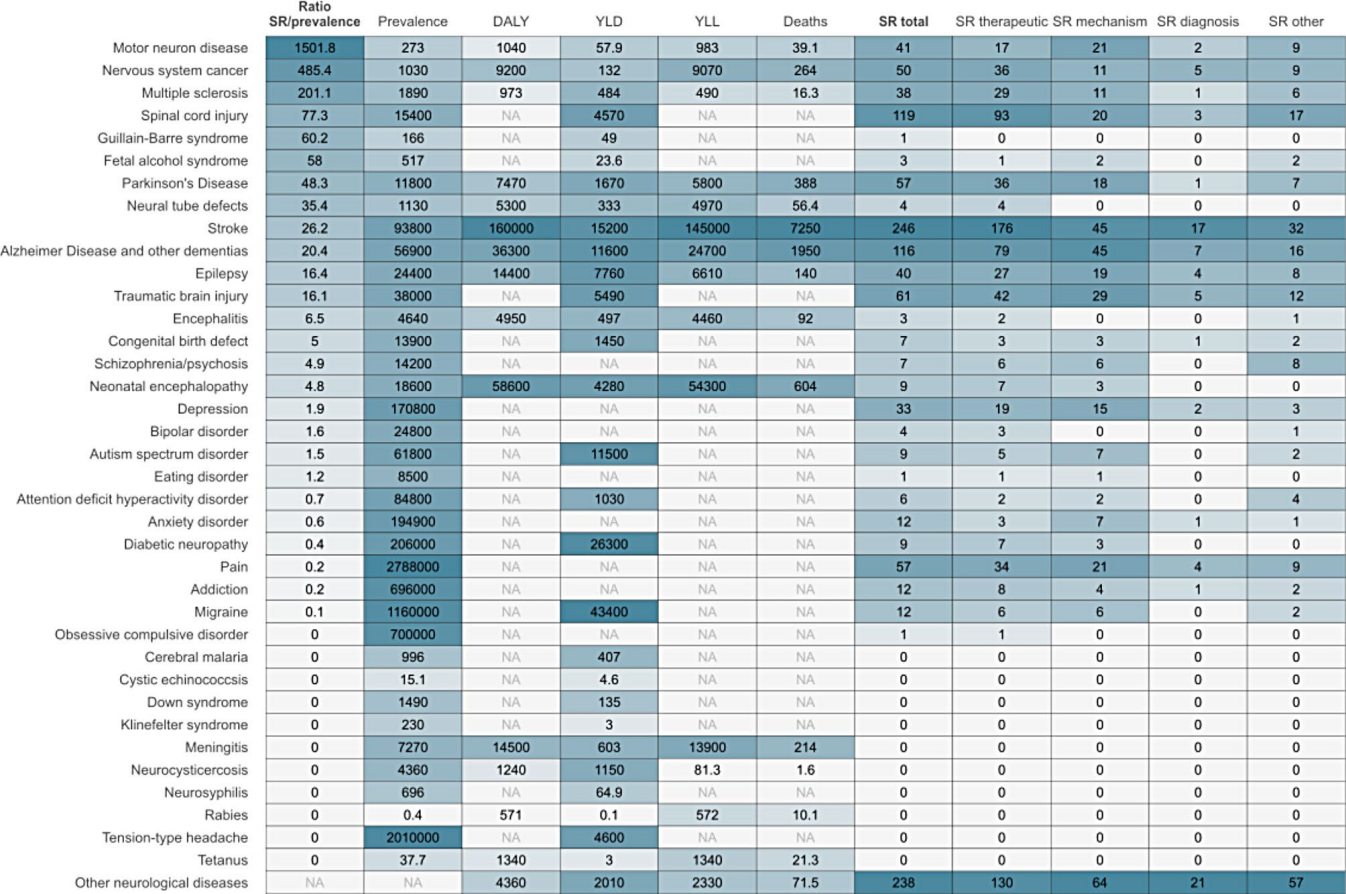
Disease burden metrics and coverage of preclinical systematic reviews. This table presents disease burden metrics, including global disease prevalence (in thousands), disability-adjusted life-years (DALY), years lived with disability (YLD), years of life lost (YLL), and deaths. The metrics are based on disease categories from the Global Burden of Disease study for neurological [28] and mental disorders [29]. Additionally, the table includes the number of systematic reviews per disease, further categorized by focal topics: therapeutic interventions, pathophysiology and mechanisms, diagnostic tools, and biomarkers, and other. The first column displays the ratio of systematic reviews to disease prevalence (multiplied by 10,000) to indicate the relative over- or under-representation of systematic reviews for each disease. *Abbreviations: DALYs = disability-adjusted life-years; SR*,* systematic review; YLDs = years lived with disability; YLLs = years of life lost*

The most frequently covered diseases were stroke (*n* = 246, 18%), spinal cord injury (*n* = 119, 9%), Alzheimer’s disease (*n* = 116, 9%), traumatic brain injury (*n* = 61, 4%), pain and Parkinson’s disease (*n* = 57 each, 4%), nervous system cancer (*n* = 50, 4%), motor neuron disease (*n* = 41, 3%), epilepsy (*n* = 40, 3%), multiple sclerosis (*n* = 38, 3%), and depression (*n* = 33, 2%) (Fig. 3). The majority of systematic reviews focused on therapeutic interventions (65%), followed by studies on pathophysiological mechanisms (30%). Reviews with a primary focus on diagnostic approaches or biomarkers were less common (6%).

Finally, when correlating the number of systematic reviews to the prevalence of neurological and psychiatric diseases using global burden of disease data, certain topics appeared relatively overrepresented. These included motor neuron diseases (e.g., amyotrophic lateral sclerosis), which had the highest systematic review-disease prevalence ratio, as well as nervous system cancer, multiple sclerosis, spinal cord injury, and Guillain-Barré syndrome (Fig. 3). Conversely, many common psychiatric diseases such as depression, bipolar disorders, autism spectrum disorder, eating disorders, attention deficit hyperactivity disorder, anxiety disorders, and addiction, as well as conditions like diabetic neuropathy, pain, and migraine, were underrepresented. Notably, some prevalent disorders, including obsessive-compulsive disorder, Down syndrome, meningitis, and tension-type headache, had no identified preclinical systematic reviews at all.

### Geography of systematic reviews

**Fig. 4:**
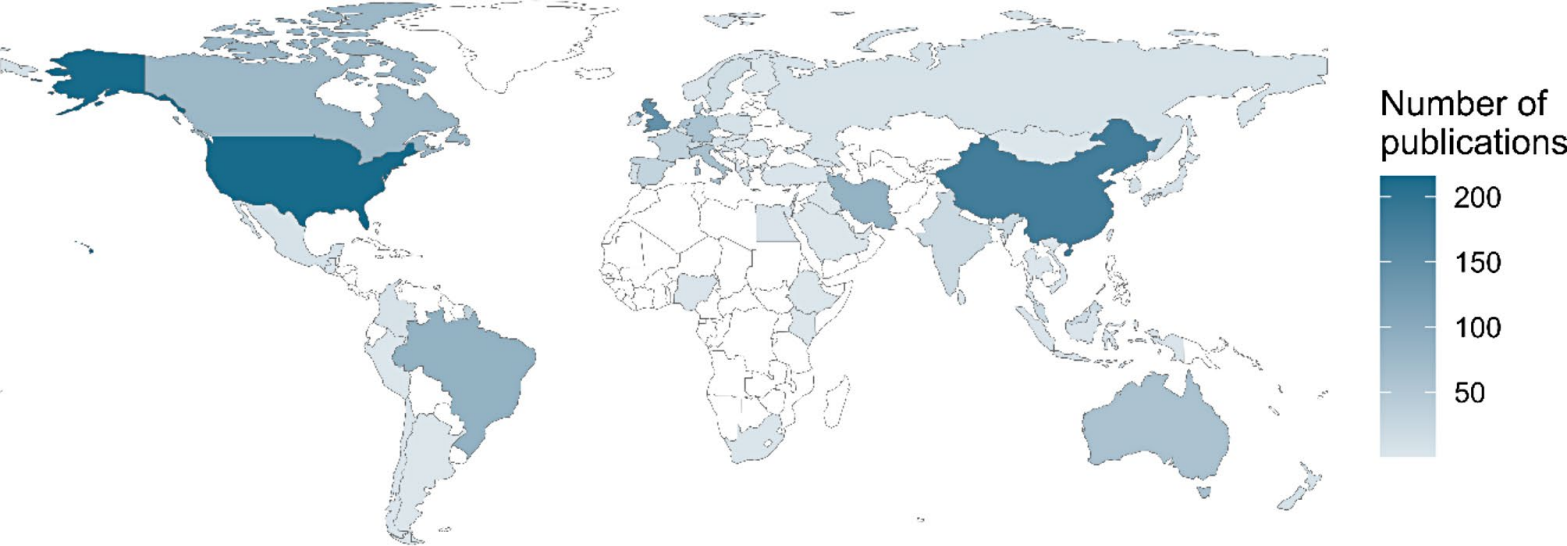
World heat map of countries publishing animal systematic reviews. Animal systematic reviews stemmed from 64 countries across all continents

Included systematic reviews stemmed from 64 countries across all continents (Fig. 4).

Europe emerged as the most prolific producer of systematic reviews (*n* = 507, 37% of total). The top three countries within Europe account for 54% of this output, with the UK (*n* = 144), the Netherlands (*n* = 62), and Italy (*n* = 61) leading the way. Asia was the second most prolific continent (*n* = 361, 26%). Notably, half of Asia’s publications originate from China (*n* = 182), with Iran also being a prolific country (*n* = 87). The third most prolific continent was North America (*n* = 301, 22%), with reviews mostly published be the United States (*n* = 216, 72% of North American publications) and Canada (*n* = 78, 26%). There were also systematic reviews from South America (*n* = 101, 8%, predominantly represented by Brazil, *n* = 89), Australia & Oceania (*n* = 69 studies, 5%), and Africa (*n* = 13, 1%, mostly from Egypt, Nigeria, and South Africa). Median annual growth rates for Europe, Asia, North America, South America, and Australia and Oceania were + 20%, + 44%, + 21%, + 38%, and + 16%, respectively.

**Fig. 5:**
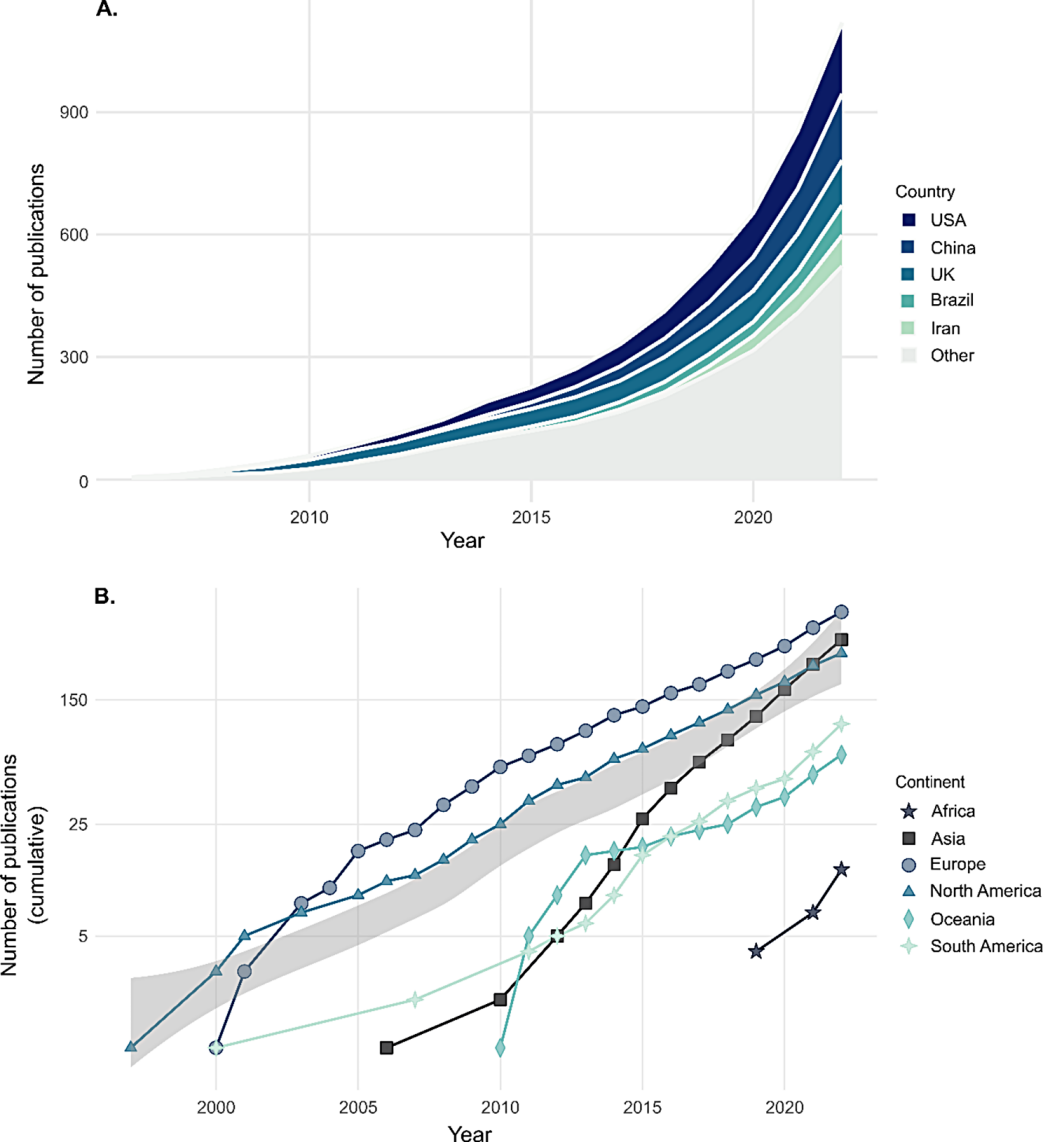
Prolific countries publishing animal systematic reviews. The 5 most prolific countries were the USA, China, UK, Brazil, and Iran (**A**). Europe was the most prolific continent in the production of systematic review (**B**). The median global growth of systematic reviews was 26%

The most prolific countries, collectively covering nearly 50% of all SR, were the USA (*n* = 216, 16% of global publications), China (*n* = 182, 13%), the UK (*n* = 144, 11%), Brazil (*n* = 89, 7%), Iran (*n* = 87, 6%), and Canada (*n* = 78, 6%) (Fig. 5A). Median annual growth rates for these countries were + 22%, + 42%, + 14%, + 33%, + 70%, and + 19%, respectively (Fig. 5B). This pattern remained consistent when analysing only systematic reviews without meta-analyses (Supplementary Figs. 1 and 2).

### Quality of systematic reviews globally and over time

The automated quality mining function performed well in a random sample of the included references, with F1-scores well above 0.8 for most items (expect for whether the existence of a preregistered study protocol was reported in the systematic review, F1-score = 0.72) (Supplementary Table 2).

**Fig. 6:**
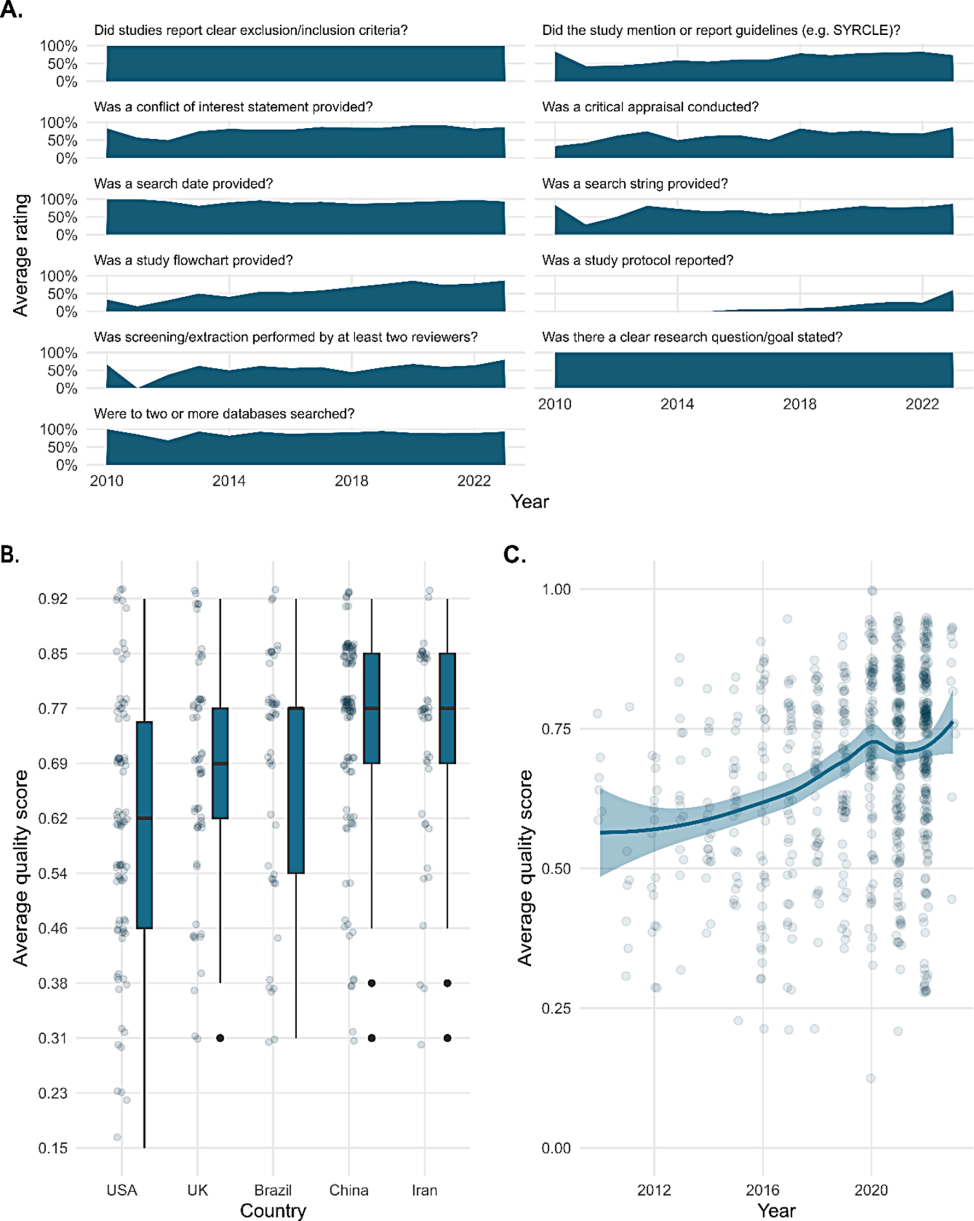
Quality of animal systematic reviews. Quality of animal systematic reviews overall (**A**), per scored item (**B**), and for the most prolific countries publishing animal systematic reviews (**C**). There is a significant increase in systematic review quality over time. The countries show no statistically significant quality differences

The overall median quality score of systematic reviews (with “1” and “0” respectively signifying that all or none of the eleven quality items were fulfilled) was 0.818 (range 0.182–1) (Fig. 6A). The three most commonly fulfilled items, all related to reporting quality, were a clear statement of a research question (Fulfilled by 100% of included systematic reviews), the reporting of in- and exclusion criteria (100%), and reporting of a search date (93%). The three least checked items, all related to methodological quality, were the conduction of a critical appraisal of included primary studies (69%), the screening/extraction being conducted by two or more reviewers (59%), and the reporting of a study protocol (18%) (Fig. 6B).

Over time, the overall quality of systematic reviews significantly improved, with a median score of 0.51 (range 0.47–0.67) in the publication date range 2010–2012 and a median score of 0.71 (range 0.70–0.72) for 2020–2022 (Wilcoxon test, p-value < 0.001). Items with a notable increase over time were the reporting of a study protocol, and presentation of a flow chart for study inclusion. Before 2015, there was no mention of a study protocol in any SR. After 2015, there was an increase in systematic reviews reporting such a protocol with currently around 40% of systematic reviews fulfilling this criterium. None of the items showed a decrease.

The median quality score for the 5 most prolific countries were 0.73, 0.82, 0.73, 0.82, and 0.82 for the USA, China, UK, Brazil, and Iran, respectively. There was no statistically significant difference in the quality score between countries. All these countries show a quality increase over time (Fig. 6C). A quality score per country is presented in Supplementary Table 3.

Systematic reviews with a reported study protocol had higher quality scores compared to those without such protocols (0.92, ± SD 0.12 versus 0.79 ± 0.19, t-test: *p* < 0.001, excluding the item protocol from the analysis).

## Discussion

### Main findings

We aimed at summarizing the landscape of animal systematic reviews in neuroscience, including their global distribution, quality, and temporal trends. We found that (1) animal systematic reviews are increasingly employed, covering a broad range of topics, with many of them addressing translational research questions, (2) The most prolific countries in publishing systematic reviews are the USA, China, the UK, Brazil, and Iran, and (3) The quality of systematic reviews is generally high, increasing over time, and notably, there is no difference in quality between countries.

### Findings in the context of existing evidence

The increasing utilization of animal systematic reviews reflects a growing trend previously noted in older studies [30, 31]. This rise corresponds with the expanding publication of primary animal studies [18]. The value of systematic reviews, and meta-analyses where applicable, lies in their capacity to evaluate evidence while minimizing the risk of bias in the research review process. Their adoption also promotes transparency, assists in identifying gaps in evidence, and aids in shaping future research directions and study designs.

In neuroscience, animal systematic reviews often focus on translational research, bridging animal and human studies. Stroke is the most frequently covered topic, despite its historically limited success in clinical translation [32, 33]. This focus is likely influenced by the early efforts of the CAMARADES consortium, which prioritized translational stroke research [9]. Other commonly reviewed topics include brain tumours and neurodegenerative diseases, which have also faced challenges in clinical translation [34, 35], as well as spinal cord injury, traumatic brain injury, and depression. Diseases with more successful translational outcomes, such as multiple sclerosis [8], epilepsy [36], and pain [37], are also represented in systematic reviews. Consistent with the translational emphasis of these reviews, most focus on potential therapeutic approaches, while those addressing diagnostic approaches remain relatively rare. This scarcity on diagnostics could reflect a general lack of robust animal models for diagnostic tool development, as these tools often require human-specific biomarkers and validation in clinical settings.

There are notable disparities in the coverage of diseases by systematic reviews, with some diseases being overrepresented and others showing clear gaps. Motor neuron diseases such as amyotrophic lateral sclerosis are relatively overrepresented when compared to their prevalence. This overrepresentation is likely driven in part by the high fatality rate of this relatively rare disease and the lack of available therapeutic options [38, 39]. Surprisingly, many common psychiatric diseases, including depression, bipolar disorders, autism spectrum disorder, eating disorders, attention deficit hyperactivity disorder, anxiety disorders, and addiction, are highly underrepresented, with only a few systematic reviews in comparison to their prevalence. One reason for this may be the perception that animal models are less reliable in mimicking complex behavioral phenotypes [40] or the high heterogeneity of underlying evidence, given the variety of animal models used to simulate specific psychiatric conditions (e.g., the chronic unpredictable mild stress model for depression and anxiety) [41]. Furthermore, several highly prevalent disorders, including obsessive-compulsive disorder, Down syndrome, meningitis, and tension-type headache, had no identified preclinical systematic reviews. This could reflect a lack of robust or widely accepted animal models for these conditions.

The most prolific publishers of animal systematic reviews are the USA, China, and the UK, aligning with their overall research output and funding [42, 43]. Interestingly, Brazil and Iran also emerge as leading contributors to animal systematic reviews, which contrasts with their lower ranking in the publication of systematic reviews in general [44]. This may be indicative of a stronger focus on preclinical research within these countries. Notably, Brazil’s involvement with BRISA and the CAMARADES network likely supports their capability in this area.

The quality of systematic reviews has generally been high and has improved over time. Most reviews present clear research questions and define inclusion and exclusion criteria effectively. However, there is a notable gap in the critical appraisal of included studies and the publication of pre-study protocols. This aligns with earlier findings that placed the quality of animal systematic reviews between those dealing with in vitro and patient data [45]. Interestingly, we found no statistically significant quality differences across countries, consistent with recent comparisons of clinical systematic reviews from China and the USA [46]. This observation challenges previous perceptions of varying quality among meta-analyses, particularly from China [13, 47], suggesting that the adoption of formal reporting guidelines, like the preclinical PRISMA extension [23, 48], has positively impacted overall quality.

While animal systematic reviews can provide insights into disease mechanisms and translational research, we recognize the global shift towards reducing animal models in favor of new approach, or non-animal, methodologies (NAMs) [49, 50]. Nonetheless, systematic reviews of animal studies remain important due to the extensive body of existing animal research [18]. Additionally, systematic reviews can also incorporate data from non-animal evidence sources, making them a good tool to also address evidence from non-animal studies such as in vitro studies [3].

### Limitations

Our study has certain limitations. First, our relatively broad definition of systematic reviews included some studies that do not strictly adhere to the Cochrane Collaboration’s definition, with some reviews being in a more narrative fashion. This may lead to a slight underestimation of certain quality criteria; however, we believe this applies to only a small number of reviews. Second, we assessed quality based on reported information, which may not always accurately reflect the actual execution quality of the systematic reviews and meta-analyses. Finally, although we conducted a dual screening of the studies based on their abstracts, we did not perform a thorough examination of the full texts to confirm their eligibility for inclusion in our study. Despite this, we believe that the abstracts generally offer enough information to determine whether a study qualifies as a systematic review.

### Strengths

Our study has the following strengths: First, with 1,358 included systematic reviews, it has a large sample size and thus offers a good foundation for analysis. Second, we developed and validated an automated tool for quality assessment, achieving high reliability and enabling efficient, large-scale evaluations. Third, our comprehensive mapping of topics and geographical trends provides insights into the focus areas and global distribution of animal research evidence synthesis.

### Recommendations

Based on our findings, we call for the following two actions: First, an effort to strengthen preclinical evidence synthesis for psychiatric diseases, including depression, bipolar disorders, autism spectrum disorder, eating disorders, attention deficit hyperactivity disorder, and anxiety disorders but also for diseases like meningitis. Thus, efforts should be undertaken to communicate these findings to target stakeholders to improve implementation in practice, e.g., at conferences or scientific journals [51, 52]. Second, while the overall rigor of included systematic reviews and meta-analyses is relatively high, there is a need to emphasize pre-registration of study protocols and critical appraisal of included studies—two key strategies to mitigate common biases in systematic reviews [3]. This could be achieved by integrating these elements into teaching initiatives, such as summer schools [53] or online courses, and emphasizing them in methodological papers on systematic reviews.

## Conclusions

With the rise of animal systematic reviews, and although generally of high quality, we identify concrete topical and quality targets to further enhance animal systematic reviews. Among them the recommended a priori publication of a study protocol. Such measures can contribute to the effective translation of animal research findings to clinical applications.

## Electronic supplementary material

Below is the link to the electronic supplementary material.

Supplementary Material 1

Supplementary Material 2

## Data Availability

All data and code that support the findings of this study are available om the Open Science Framework at https://osf.io/wx5ta/. For any questions regarding data, meta-data, and analysis, contact the corresponding author BVI.
