# Supplementary material for: Quality, topics, and demographic trends of animal systematic reviews - an umbrella review
Source: J Transl Med. 2025 Jan 6;23:21. doi: 10.1186/s12967-024-05992-0 (PMC11702210; doi:10.1186/s12967-024-05992-0)
Supplement: Supplementary file 1 — Supplementary Material 1 [file 12967_2024_5992_MOESM1_ESM.pdf]

Embase

Embase session results (27 Jan 2023)

No.	Query	Results
#11	#3 AND #9 NOT [26-01-2023]/sd	16424
#10	#3 AND #9	16449
#9	#7 OR #8	16669517
#8	'animal experiment'/exp OR 'animal model'/exp OR 'experimental animal'/exp OR 'transgenic animal'/exp OR 'male animal'/exp OR 'female animal'/exp OR 'juvenile animal'/exp OR 'animal'/de OR 'chordata'/de OR 'vertebrate'/de OR 'tetrapod'/de OR 'fish'/exp OR 'amniote'/de OR 'amphibia'/exp OR 'mammal'/de OR 'reptile'/exp OR 'sauropsid'/exp OR 'therian'/de OR 'monotremate'/exp OR 'placental mammals'/de OR 'marsupial'/exp OR 'euarchontoglires'/de OR 'afrotheria'/exp OR 'boreoeutheria'/exp OR 'laurasiatheria'/exp OR 'xenarthra'/exp OR 'primate'/de OR 'dermoptera'/exp OR 'glires'/exp OR 'scandentia'/exp OR 'haplorhini'/de OR 'prosimian'/exp OR 'simian'/de OR 'tarsiiform'/exp OR 'catarrhini'/de OR 'platyrrhini'/exp OR 'ape'/de OR 'cercopithecidae'/exp OR 'hominid'/de OR 'hylobatidae'/exp OR 'chimpanzee'/exp OR 'gorilla'/exp OR 'orang utan'/exp OR animal:ab,ti OR animals:ab,ti OR pisces:ab,ti OR fish:ab,ti OR fishes:ab,ti OR catfish:ab,ti OR catfishes:ab,ti OR sheatfish:ab,ti OR silurus:ab,ti OR arius:ab,ti OR heteropneustes:ab,ti OR clarias:ab,ti OR gariepinus:ab,ti OR 'fathead minnow':ab,ti OR 'fathead minnows':ab,ti OR pimephales:ab,ti OR promelas:ab,ti OR cichlidae:ab,ti OR trout:ab,ti OR trouts:ab,ti OR char:ab,ti OR chars:ab,ti OR salvelinus:ab,ti OR salmo:ab,ti OR oncorhynchus:ab,ti OR guppy:ab,ti OR guppies:ab,ti OR millionfish:ab,ti OR poecilia:ab,ti OR goldfish:ab,ti OR goldfishes:ab,ti OR carassius:ab,ti OR auratus:ab,ti OR mullet:ab,ti OR mullets:ab,ti OR mugil:ab,ti OR curema:ab,ti OR shark:ab,ti OR sharks:ab,ti OR cod:ab,ti OR cods:ab,ti OR gadus:ab,ti OR morhua:ab,ti OR carp:ab,ti OR carps:ab,ti OR cyprinus:ab,ti OR carpio:ab,ti OR killifish:ab,ti OR eel:ab,ti OR eels:ab,ti OR anguilla:ab,ti OR zander:ab,ti OR sander:ab,ti OR lucioperca:ab,ti OR stizostedion:ab,ti OR turbot:ab,ti OR turbots:ab,ti OR psetta:ab,ti OR flatfish:ab,ti OR flatfishes:ab,ti OR plaice:ab,ti OR pleuronectes:ab,ti OR platessa:ab,ti OR tilapia:ab,ti OR tilapias:ab,ti OR	8545662

oreochromis:ab,ti OR sarotherodon:ab,ti OR 'common sole':ab,ti OR 'dover sole':ab,ti OR solea:ab,ti OR zebrafish:ab,ti OR zebrafishes:ab,ti OR danio:ab,ti OR rerio:ab,ti OR seabass:ab,ti OR dicentrarchus:ab,ti OR labrax:ab,ti OR morone:ab,ti OR lamprey:ab,ti OR lampreys:ab,ti OR petromyzon:ab,ti OR pumpkinseed:ab,ti OR pumpkinseeds:ab,ti OR lepomis:ab,ti OR gibbosus:ab,ti OR herring:ab,ti OR clupea:ab,ti OR harengus:ab,ti OR amphibia:ab,ti OR amphibian:ab,ti OR amphibians:ab,ti OR anura:ab,ti OR salientia:ab,ti OR frog:ab,ti OR frogs:ab,ti OR rana:ab,ti OR toad:ab,ti OR toads:ab,ti OR bufo:ab,ti OR xenopus:ab,ti OR laevis:ab,ti OR bombina:ab,ti OR epidalea:ab,ti OR calamita:ab,ti OR salamander:ab,ti OR salamanders:ab,ti OR newt:ab,ti OR newts:ab,ti OR triturus:ab,ti OR reptilia:ab,ti OR reptile:ab,ti OR reptiles:ab,ti OR 'bearded dragon':ab,ti OR pogona:ab,ti OR vitticeps:ab,ti OR iguana:ab,ti OR iguanas:ab,ti OR lizard:ab,ti OR lizards:ab,ti OR 'anguis fragilis':ab,ti OR turtle:ab,ti OR turtles:ab,ti OR snakes:ab,ti OR snake:ab,ti OR aves:ab,ti OR bird:ab,ti OR birds:ab,ti OR quail:ab,ti OR quails:ab,ti OR coturnix:ab,ti OR bobwhite:ab,ti OR colinus:ab,ti OR virginianus:ab,ti OR poultry:ab,ti OR poultries:ab,ti OR fowl:ab,ti OR fowls:ab,ti OR chicken:ab,ti OR chickens:ab,ti OR gallus:ab,ti OR 'zebra finch':ab,ti OR taeniopygia:ab,ti OR guttata:ab,ti OR canary:ab,ti OR canaries:ab,ti OR serinus:ab,ti OR canaria:ab,ti OR parakeet:ab,ti OR parakeets:ab,ti OR grasskeet:ab,ti OR parrot:ab,ti OR parrots:ab,ti OR psittacine:ab,ti OR psittacines:ab,ti OR shelduck:ab,ti OR tadorna:ab,ti OR goose:ab,ti OR geese:ab,ti OR branta:ab,ti OR leucopsis:ab,ti OR woodlark:ab,ti OR lullula:ab,ti OR flycatcher:ab,ti OR ficedula:ab,ti OR hypoleuca:ab,ti OR dove:ab,ti OR doves:ab,ti OR geopelia:ab,ti OR cuneata:ab,ti OR duck:ab,ti OR ducks:ab,ti OR greylag:ab,ti OR graylag:ab,ti OR anser:ab,ti OR harrier:ab,ti OR 'circus pygargus':ab,ti OR 'red knot':ab,ti OR 'great knot':ab,ti OR calidris:ab,ti OR canutus:ab,ti OR godwit:ab,ti OR limosa:ab,ti OR lapponica:ab,ti OR meleagris:ab,ti OR gallopavo:ab,ti OR jackdaw:ab,ti OR corvus:ab,ti OR monedula:ab,ti OR ruff:ab,ti OR philomachus:ab,ti OR pugnax:ab,ti OR lapwing:ab,ti OR peewit:ab,ti OR plover:ab,ti OR vanellus:ab,ti OR swan:ab,ti OR cygnus:ab,ti OR columbianus:ab,ti OR bewickii:ab,ti OR gull:ab,ti OR chroicocephalus:ab,ti OR ridibundus:ab,ti OR albifrons:ab,ti OR 'great tit':ab,ti OR parus:ab,ti OR aythya:ab,ti OR fuligula:ab,ti OR streptopelia:ab,ti OR risoria:ab,ti OR spoonbill:ab,ti OR platalea:ab,ti OR leucorodia:ab,ti OR blackbird:ab,ti OR turdus:ab,ti OR merula:ab,ti OR 'blue tit':ab,ti OR cyanistes:ab,ti OR pigeon:ab,ti OR pigeons:ab,ti OR columba:ab,ti OR pintail:ab,ti OR anas:ab,ti OR starling:ab,ti OR sturnus:ab,ti OR owl:ab,ti OR 'athene noctua':ab,ti OR pochard:ab,ti OR ferina:ab,ti OR cockatiel:ab,ti OR nymphicus:ab,ti OR hollandicus:ab,ti OR skylark:ab,ti OR alauda:ab,ti OR tern:ab,ti OR sterna:ab,ti OR teal:ab,ti OR crecca:ab,ti OR oystercatcher:ab,ti OR haematopus:ab,ti OR ostralegus:ab,ti OR shrew:ab,ti OR shrews:ab,ti OR sorex:ab,ti OR araneus:ab,ti OR crocidura:ab,ti OR russula:ab,ti OR 'european mole':ab,ti OR

talpa:ab,ti OR chiroptera:ab,ti OR bat:ab,ti OR bats:ab,ti OR eptesicus:ab,ti OR serotinus:ab,ti OR myotis:ab,ti OR dasycneme:ab,ti OR daubentonii:ab,ti OR pipistrelle:ab,ti OR pipistrellus:ab,ti OR cat:ab,ti OR cats:ab,ti OR felis:ab,ti OR catus:ab,ti OR feline:ab,ti OR dog:ab,ti OR dogs:ab,ti OR canis:ab,ti OR canine:ab,ti OR canines:ab,ti OR otter:ab,ti OR otters:ab,ti OR lutra:ab,ti OR badger:ab,ti OR badgers:ab,ti OR meles:ab,ti OR fitchew:ab,ti OR fitch:ab,ti OR foumart:ab,ti OR foulmart:ab,ti OR ferrets:ab,ti OR ferret:ab,ti OR polecat:ab,ti OR polecats:ab,ti OR mustela:ab,ti OR putorius:ab,ti OR weasel:ab,ti OR weasels:ab,ti OR fox:ab,ti OR foxes:ab,ti OR vulpes:ab,ti OR 'common seal':ab,ti OR phoca:ab,ti OR vitulina:ab,ti OR 'grey seal':ab,ti OR halichoerus:ab,ti OR horse:ab,ti OR horses:ab,ti OR equus:ab,ti OR equine:ab,ti OR equidae:ab,ti OR donkey:ab,ti OR donkeys:ab,ti OR mule:ab,ti OR mules:ab,ti OR pig:ab,ti OR pigs:ab,ti OR swine:ab,ti OR swines:ab,ti OR hog:ab,ti OR hogs:ab,ti OR boar:ab,ti OR boars:ab,ti OR porcine:ab,ti OR piglet:ab,ti OR piglets:ab,ti OR sus:ab,ti OR scrofa:ab,ti OR llama:ab,ti OR llamas:ab,ti OR lama:ab,ti OR glama:ab,ti OR deer:ab,ti OR deers:ab,ti OR cervus:ab,ti OR elaphus:ab,ti OR cow:ab,ti OR cows:ab,ti OR 'bos taurus':ab,ti OR 'bos indicus':ab,ti OR bovine:ab,ti OR bull:ab,ti OR bulls:ab,ti OR cattle:ab,ti OR bison:ab,ti OR bisons:ab,ti OR sheep:ab,ti OR sheeps:ab,ti OR 'ovis aries':ab,ti OR ovine:ab,ti OR lamb:ab,ti OR lambs:ab,ti OR mouflon:ab,ti OR mouflons:ab,ti OR goat:ab,ti OR goats:ab,ti OR capra:ab,ti OR caprine:ab,ti OR chamois:ab,ti OR rupicapra:ab,ti OR leporidae:ab,ti OR lagomorpha:ab,ti OR lagomorph:ab,ti OR rabbit:ab,ti OR rabbits:ab,ti OR oryctolagus:ab,ti OR cuniculus:ab,ti OR laprine:ab,ti OR hares:ab,ti OR lepus:ab,ti OR rodentia:ab,ti OR rodent:ab,ti OR rodents:ab,ti OR murinae:ab,ti OR mouse:ab,ti OR mice:ab,ti OR mus:ab,ti OR musculus:ab,ti OR murine:ab,ti OR woodmouse:ab,ti OR apodemus:ab,ti OR rat:ab,ti OR rats:ab,ti OR rattus:ab,ti OR norvegicus:ab,ti OR 'guinea pig':ab,ti OR 'guinea pigs':ab,ti OR cavia:ab,ti OR porcellus:ab,ti OR hamster:ab,ti OR hamsters:ab,ti OR mesocricetus:ab,ti OR cricetulus:ab,ti OR cricetus:ab,ti OR gerbil:ab,ti OR gerbils:ab,ti OR jird:ab,ti OR jirds:ab,ti OR meriones:ab,ti OR unguiculatus:ab,ti OR jerboa:ab,ti OR jerboas:ab,ti OR jaculus:ab,ti OR chinchilla:ab,ti OR chinchillas:ab,ti OR beaver:ab,ti OR beavers:ab,ti OR 'castor fiber':ab,ti OR 'castor canadensis':ab,ti OR sciuridae:ab,ti OR squirrel:ab,ti OR squirrels:ab,ti OR sciurus:ab,ti OR chipmunk:ab,ti OR chipmunks:ab,ti OR marmot:ab,ti OR marmots:ab,ti OR marmota:ab,ti OR suslik:ab,ti OR susliks:ab,ti OR spermophilus:ab,ti OR cynomys:ab,ti OR cottonrat:ab,ti OR cottonrats:ab,ti OR sigmodon:ab,ti OR vole:ab,ti OR voles:ab,ti OR microtus:ab,ti OR myodes:ab,ti OR glareolus:ab,ti OR primate:ab,ti OR primates:ab,ti OR prosimian:ab,ti OR prosimians:ab,ti OR lemur:ab,ti OR lemurs:ab,ti OR lemuridae:ab,ti OR loris:ab,ti OR 'bush baby':ab,ti OR 'bush babies':ab,ti OR bushbaby:ab,ti OR bushbabies:ab,ti OR galago:ab,ti OR galagos:ab,ti OR anthropoidea:ab,ti OR anthropoids:ab,ti OR simian:ab,ti OR

	simians:ab,ti OR monkey:ab,ti OR monkeys:ab,ti OR marmoset:ab,ti OR marmosets:ab,ti OR callithrix:ab,ti OR cebuella:ab,ti OR tamarin:ab,ti OR tamarins:ab,ti OR saguinus:ab,ti OR leontopithecus:ab,ti OR 'squirrel monkey':ab,ti OR 'squirrel monkeys':ab,ti OR saimiri:ab,ti OR 'night monkey':ab,ti OR 'night monkeys':ab,ti OR 'owl monkey':ab,ti OR 'owl monkeys':ab,ti OR douroucoulis:ab,ti OR aotus:ab,ti OR 'spider monkey':ab,ti OR 'spider monkeys':ab,ti OR ateles:ab,ti OR baboon:ab,ti OR baboons:ab,ti OR papio:ab,ti OR 'rhesus monkey':ab,ti OR macaque:ab,ti OR macaca:ab,ti OR mulatta:ab,ti OR cynomolgus:ab,ti OR fascicularis:ab,ti OR 'green monkey':ab,ti OR 'green monkeys':ab,ti OR chlorocebus:ab,ti OR vervet:ab,ti OR vervets:ab,ti OR pygerythrus:ab,ti OR hominoidea:ab,ti OR ape:ab,ti OR apes:ab,ti OR hylobatidae:ab,ti OR gibbon:ab,ti OR gibbons:ab,ti OR siamang:ab,ti OR siamangs:ab,ti OR nomascus:ab,ti OR symphalangus:ab,ti OR hominidae:ab,ti OR orangutan:ab,ti OR orangutans:ab,ti OR pongo:ab,ti OR chimpanzee:ab,ti OR chimpanzees:ab,ti OR 'pan troglodytes':ab,ti OR bonobo:ab,ti OR bonobos:ab,ti OR 'pan paniscus':ab,ti OR gorilla:ab,ti OR gorillas:ab,ti OR troglodytes:ab,ti	
#7	'preclinical study'/de OR 'animal experiment'/exp OR 'in vitro study'/exp OR 'nonhuman'/exp OR (((cell OR tissue OR organ) NEAR/12 (culture* OR experiment* OR stud* OR model* OR line)):ti,ab,kw) OR cell:de OR preclinical:ti,ab,kw OR 'pre clinical':ti,ab,kw OR 'in vitro':ti,ab,kw OR 'ex vivo':ti,ab,kw OR nonhuman:ti,ab,kw OR 'non human':ti,ab,kw	13930347
#6	#3 AND #4 NOT [26-01-2023]/sd	69886
#5	#3 AND #4	69968
#4	'human'/exp NOT ((((cell OR tissue OR organ) NEAR/12 (culture* OR experiment* OR stud* OR model* OR line)):ti,ab,kw) OR cell:de OR preclinical:ti,ab,kw OR 'pre clinical':ti,ab,kw OR 'in vitro':ti,ab,kw OR 'ex vivo':ti,ab,kw)	20632755
#3	#1 AND #2 NOT [conference abstract]/lim AND [english]/lim	81649
#2	'meta analysis'/exp OR 'systematic review'/exp OR (systematic:ti AND review*:ti) OR (meta:ti AND analys*:ti) OR metaanalys*:ti OR 'meta analys*':ti	574257
#1	'neurologic disease'/exp OR 'neuroscience'/exp OR neuroscien*:ti,kw OR neuroanatom*:ti,kw OR neurobiolog*:ti,kw OR neurochemi*:ti,kw OR	4731853

neuroeconomic*:ti,kw OR neuroendocrin*:ti,kw OR neurolog*:ti,kw OR neuropatholog*:ti,kw OR neuropharmacolog*:ti,kw OR neurophysiolog*:ti,kw OR neuropsychiatr*:ti,kw OR neuropsyc*:ti,kw OR psychiatr*:ti,kw OR neurotraumatolog*:ti,kw

Copyright © 2023 Elsevier Limited except certain content provided by third parties.

Embase is a trade mark of Elsevier Life Sciences IP Limited.

Thursday, January 26, 2023 3:14:51 PM

#	Query	Limiters/Expanders	Last Run Via	Results
S12	S3 AND S10	Limiters - English Language<br>Expanders - Apply equivalent subjects<br>Search modes - Find all my search terms	Interface - EBSCOhost Research Databases<br>Search Screen - Advanced Search<br>Database - MEDLINE	3,666
S11	S3 AND S10	Expanders - Apply equivalent subjects<br>Search modes - Find all my search terms	Interface - EBSCOhost Research Databases<br>Search Screen - Advanced Search<br>Database - MEDLINE	3,723
S10	S8 OR S9	Expanders - Apply equivalent subjects<br>Search modes - Find all my search terms	Interface - EBSCOhost Research Databases<br>Search Screen - Advanced Search<br>Database - MEDLINE	10,520,662
S9	(MH "animal experimentation+" OR MH "models, animal+" OR MH "invertebrates+" OR MH "Animals" OR MH "animal population groups+" OR MH "chordata" OR MH "chordata, nonvertebrate+" OR MH "vertebrates" OR MH "amphibians+" OR MH "birds+" OR MH "fishes+" OR MH "reptiles+" OR MH "mammals" OR MH "primates" OR MH "artiodactyla+" OR MH "carnivora+" OR MH "cetacea+" OR MH "chiroptera+" OR MH "elephants+" OR MH "hyraxes+" OR MH "insectivora+" OR MH "lagomorpha+" OR MH "marsupialia+" OR MH "monotremata+" OR MH "perissodactyla+" OR MH "rodentia+" OR MH	Expanders - Apply equivalent subjects<br>Search modes - Find all my search terms	Interface - EBSCOhost Research Databases<br>Search Screen - Advanced Search<br>Database - MEDLINE	7,673,120

"scandentia+" OR MH "sirenia+" OR MH "xenarthra+" OR MH "haplorhini" OR MH "strepsirhini+" OR MH "platyrrhini+" OR MH "tarsii+" OR MH "catarrhini" OR MH "cercopithecidae+" OR MH "hylobatidae+" OR MH "hominidae" OR MH "gorilla gorilla+" OR MH "pan paniscus+" OR MH "pan troglodytes+" OR MH "pongo pygmaeus+")OR ((TI(animals OR animal OR mice OR mus OR mouse OR murine OR woodmouse OR rats OR rat OR murinae OR muridae OR cottonrat OR cottonrats OR hamster OR hamsters OR cricetinae OR rodentia OR rodent OR rodents OR pigs OR pig OR swine OR swines OR piglets OR piglet OR boar OR boars OR "sus scrofa" OR ferrets OR ferret OR polecat OR polecats OR "mustela putorius" OR "guinea pigs" OR "guinea pig" OR cavia OR callithrix OR marmoset OR marmosets OR cebuella OR hapale OR octodon OR chinchilla OR chinchillas OR gerbillinae OR gerbil OR gerbils OR jird OR jirds OR merione OR meriones OR rabbits OR rabbit OR hares OR hare OR diptera OR flies OR fly OR dipteral OR drosophila OR drosophilidae OR cats OR cat OR carus OR felis OR nematoda OR nematode OR nematodes OR sipunculida OR dogs OR dog OR canine OR canines OR canis OR sheep OR sheeps OR mouflon OR mouflons OR ovis OR goats

OR goat OR capra OR capras OR rupicapra OR rupicapras OR chamois OR haplorhini OR monkey OR monkeys OR anthropoidea OR anthropoids OR saguinus OR tamarin OR tamarins OR leontopithecus OR hominidae OR ape OR apes OR "pan paniscus" OR bonobo OR bonobos OR "pan troglodytes" OR gibbon OR gibbons OR siamang OR siamangs OR nomascus OR symphalangus OR chimpanzee OR chimpanzees OR prosimian OR prosimians OR "bush baby" OR bush babies OR galagos OR galago OR pongidae OR gorilla OR gorillas OR "pongo pygmaeus" OR orangutan OR orangutans OR lemur OR lemurs OR lemuridae OR horse OR horses OR equus OR cow OR calf OR bull OR chicken OR chickens OR gallus OR quail OR bird OR birds OR quails OR poultry OR poultries OR fowl OR fowls OR reptile OR reptilia OR reptiles OR snakes OR snake OR lizard OR lizards OR alligator OR alligators OR crocodile OR crocodiles OR turtle OR turtles OR amphibian OR amphibians OR amphibia OR frog OR frogs OR bombina OR salientia OR toad OR toads OR "epidalea calamita" OR salamander OR salamanders OR eel OR eels OR fish OR fishes OR pisces OR catfish OR catfishes OR siluriformes OR arius OR

heteropneustes OR
sheatfish OR perch OR
perches OR percidae OR
perca OR trout OR trouts
OR char OR chars OR
salvelinus OR minnow OR
cyprinidae OR carps OR
carp OR zebrafish OR
zebrafishes OR goldfish OR
goldfishes OR guppy OR
guppies OR chub OR chubs
OR tinca OR barbels OR
barbus OR pimephales OR
promelas OR "poecilia
reticulata" OR mullet OR
mullets OR eel OR eels OR
seahorse OR seahorses OR
mugil curema OR atlantic
cod OR shark OR sharks
OR catshark OR anguilla
OR salmonid OR salmonids
OR whitefish OR whitefishes
OR salmon OR salmons OR
sole OR solea OR lamprey
OR lampreys OR
pumpkinseed OR sunfish
OR sunfishes OR tilapia OR
tilapias OR turbot OR
turbots OR flatfish OR
flatfishes OR sciuridae OR
squirrel OR squirrels OR
chipmunk OR chipmunks
OR suslik OR susliks OR
vole OR voles OR lemming
OR lemmings OR muskrat
OR muskrats OR lemmus
OR otter OR otters OR
marten OR martens OR
martes OR weasel OR
badger OR badgers OR
ermine OR mink OR minks
OR sable OR sables OR
gulo OR gulos OR wolverine
OR wolverines OR mustela
OR llama OR llamas OR
alpaca OR alpacas OR
camelid OR camelids OR
guanaco OR guanacos OR
chiroptera OR chiropteras

OR bat OR bats OR fox OR foxes OR iguana OR iguanas OR xenopus laevis OR parakeet OR parakeets OR parrot OR parrots OR donkey OR donkeys OR mule OR mules OR zebra OR zebras OR shrew OR shrews OR bison OR bisons OR buffalo OR buffaloes OR deer OR deers OR bear OR bears OR panda OR pandas OR "wild hog" OR "wild boar" OR fitchew OR fitch OR beaver OR beavers OR jerboa OR jerboas OR capybara OR capybaras) OR AB(animals OR animal OR mice OR mus OR mouse OR murine OR woodmouse OR rats OR rat OR murinae OR muridae OR cottonrat OR cottonrats OR hamster OR hamsters OR cricetinae OR rodentia OR rodent OR rodents OR pigs OR pig OR swine OR swines OR piglets OR piglet OR boar OR boars OR "sus scrofa" OR ferrets OR ferret OR polecat OR polecats OR "mustela putorius" OR "guinea pigs" OR "guinea pig" OR cavia OR callithrix OR marmoset OR marmosets OR cebuella OR hapale OR octodon OR chinchilla OR chinchillas OR gerbillinae OR gerbil OR gerbils OR jird OR jirds OR merione OR meriones OR rabbits OR rabbit OR hares OR hare OR diptera OR flies OR fly OR dipteral OR drosophila OR drosophilidae OR cats OR cat OR carus OR felis OR nematoda OR nematode OR nematodes OR sipunculida OR dogs

OR dog OR canine OR canines OR canis OR sheep OR sheeps OR mouflon OR mouflons OR ovis OR goats OR goat OR capra OR capras OR rupicapra OR rupicapras OR chamois OR haplorhini OR monkey OR monkeys OR anthropoidea OR anthropoids OR saguinus OR tamarin OR tamarins OR leontopithecus OR hominidae OR ape OR apes OR "pan paniscus" OR bonobo OR bonobos OR "pan troglodytes" OR gibbon OR gibbons OR siamang OR siamangs OR nomascus OR symphalangus OR chimpanzee OR chimpanzees OR prosimian OR prosimians OR "bush baby" OR bush babies OR galagos OR galago OR pongidae OR gorilla OR gorillas OR "pongo pygmaeus" OR orangutan OR orangutans OR lemur OR lemurs OR lemuridae OR horse OR horses OR equus OR cow OR calf OR bull OR chicken OR chickens OR gallus OR quail OR bird OR birds OR quails OR poultry OR poultries OR fowl OR fowls OR reptile OR reptilia OR reptiles OR snakes OR snake OR lizard OR lizards OR alligator OR alligators OR crocodile OR crocodiles OR turtle OR turtles OR amphibian OR amphibians OR amphibia OR frog OR frogs OR bombina OR salientia OR toad OR toads OR "epidalea calamita" OR salamander OR salamanders OR eel OR

eels OR fish OR fishes OR pisces OR catfish OR catfishes OR siluriformes OR arius OR heteropneustes OR sheatfish OR perch OR perches OR percidae OR perca OR trout OR trouts OR char OR chars OR salvelinus OR minnow OR cyprinidae OR carps OR carp OR zebrafish OR zebrafishes OR goldfish OR goldfishes OR guppy OR guppies OR chub OR chubs OR tinca OR barbels OR barbus OR pimephales OR promelas OR "poecilia reticulata" OR mullet OR mullets OR eel OR eels OR seahorse OR seahorses OR mugil curema OR atlantic cod OR shark OR sharks OR catshark OR anguilla OR salmonid OR salmonids OR whitefish OR whitefishes OR salmon OR salmons OR sole OR solea OR lamprey OR lampreys OR pumpkinseed OR sunfish OR sunfishes OR tilapia OR tilapias OR turbot OR turbots OR flatfish OR flatfishes OR sciuridae OR squirrel OR squirrels OR chipmunk OR chipmunks OR suslik OR susliks OR vole OR voles OR lemming OR lemmings OR muskrat OR muskrats OR lemmus OR otter OR otters OR marten OR martens OR martes OR weasel OR badger OR badgers OR ermine OR mink OR minks OR sable OR sables OR gulo OR gulos OR wolverine OR wolverines OR mustela OR llama OR llamas OR

	alpaca OR alpacas OR camelid OR camelids OR guanaco OR guanacos OR chiroptera OR chiropteras OR bat OR bats OR fox OR foxes OR iguana OR iguanas OR xenopus laevis OR parakeet OR parakeets OR parrot OR parrots OR donkey OR donkeys OR mule OR mules OR zebra OR zebras OR shrew OR shrews OR bison OR bisons OR buffalo OR buffaloes OR deer OR deers OR bear OR bears OR panda OR pandas OR "wild hog" OR "wild boar" OR fitchew OR fitch OR beaver OR beavers OR jerboa OR jerboas OR capybara OR capybaras)) NOT SB medline)			
S8	(MH "Animal Experimentation+") OR (MH "In Vitro Techniques+") OR TI ((cell OR tissue OR organ) N12 (culture* OR experiment* OR stud* OR model* OR line)) OR AB ((cell OR tissue OR organ) N12 (culture* OR experiment* OR stud* OR model* OR line)) OR MW (cell) OR TI (preclinical OR pre-clinical OR "in vitro" OR "ex vivo" OR nonhuman OR non-human) OR AB (preclinical OR pre-clinical OR "in vitro" OR "ex vivo" OR nonhuman OR non-human)	Expanders - Apply equivalent subjects<br>Search modes - Find all my search terms	Interface - EBSCOhost Research Databases<br>Search Screen - Advanced Search<br>Database - MEDLINE	5,780,172
S7	S4 NOT S5	Limiters - English Language<br>Expanders - Apply equivalent subjects<br>Search modes - Find all my search terms	Interface - EBSCOhost Research Databases<br>Search Screen - Advanced Search<br>Database - MEDLINE	46,535
S6	S4 NOT S5	Expanders - Apply equivalent subjects	Interface - EBSCOhost Research Databases	47,659

		Search modes - Find all my search terms	Search Screen - Advanced Search<br>Database - MEDLINE	
S5	(TI ((cell OR tissue OR organ) N12 (culture* OR experiment* OR stud* OR model* OR line)) OR AB ((cell OR tissue OR organ) N12 (culture* OR experiment* OR stud* OR model* OR line)) OR MW (cell) OR TI (preclinical OR pre-clinical OR "in vitro" OR "ex vivo") OR AB (preclinical OR pre-clinical OR "in vitro" OR "ex vivo"))	Expanders - Apply equivalent subjects<br>Search modes - Find all my search terms	Interface - EBSCOhost Research Databases<br>Search Screen - Advanced Search<br>Database - MEDLINE	5,501,189
S4	S1 AND S2	Limiters - Human<br>Expanders - Apply equivalent subjects<br>Search modes - Find all my search terms	Interface - EBSCOhost Research Databases<br>Search Screen - Advanced Search<br>Database - MEDLINE	49,586
S3	S1 AND S2	Expanders - Apply equivalent subjects<br>Search modes - Find all my search terms	Interface - EBSCOhost Research Databases<br>Search Screen - Advanced Search<br>Database - MEDLINE	52,764
S2	PT ("systematic review" OR meta-analysis) OR TI ((systematic AND review*) OR (meta AND analys*) OR metaanalys* OR meta-analys*)	Expanders - Apply equivalent subjects<br>Search modes - Find all my search terms	Interface - EBSCOhost Research Databases<br>Search Screen - Advanced Search<br>Database - MEDLINE	369,445
S1	(MH "Nervous System Diseases+") OR (MH "Neurosciences+") OR TI (neuroscien* OR neuroanatom* OR neurobiolog* OR neurochemi* OR neuroeconomic* OR neuroendocrin* OR neurolog* OR neuropatholog* OR neuropharmacolog* OR neurophysiolog* OR neuropsychiatr* OR	Expanders - Apply equivalent subjects<br>Search modes - Find all my search terms	Interface - EBSCOhost Research Databases<br>Search Screen - Advanced Search<br>Database - MEDLINE	3,402,266

neuropsyc* OR psychiatr*
OR neurotraumatolog*) OR
AB (neuroscien* OR
neuroanatom* OR
neurobiolog* OR
neurochemi* OR
neuroeconomic* OR
neuroendocrin* OR
neurolog* OR
neuropatholog* OR
neuropharmacolog* OR
neurophysiolog* OR
neuropsychiatr* OR
neuropsyc* OR psychiatr*
OR neurotraumatolog*)
