# Supplementary material for: Quality, topics, and demographic trends of animal systematic reviews - an umbrella review
Source: J Transl Med. 2025 Jan 6;23:21. doi: 10.1186/s12967-024-05992-0 (PMC11702210; doi:10.1186/s12967-024-05992-0)
Supplement: Supplementary file 2 — Supplementary Material 2 [file 12967_2024_5992_MOESM2_ESM.docx]

# Supplementary data

**Supplementary Figure 1:** Prolific countries publishing animal systematic reviews (not including meta-analyses).


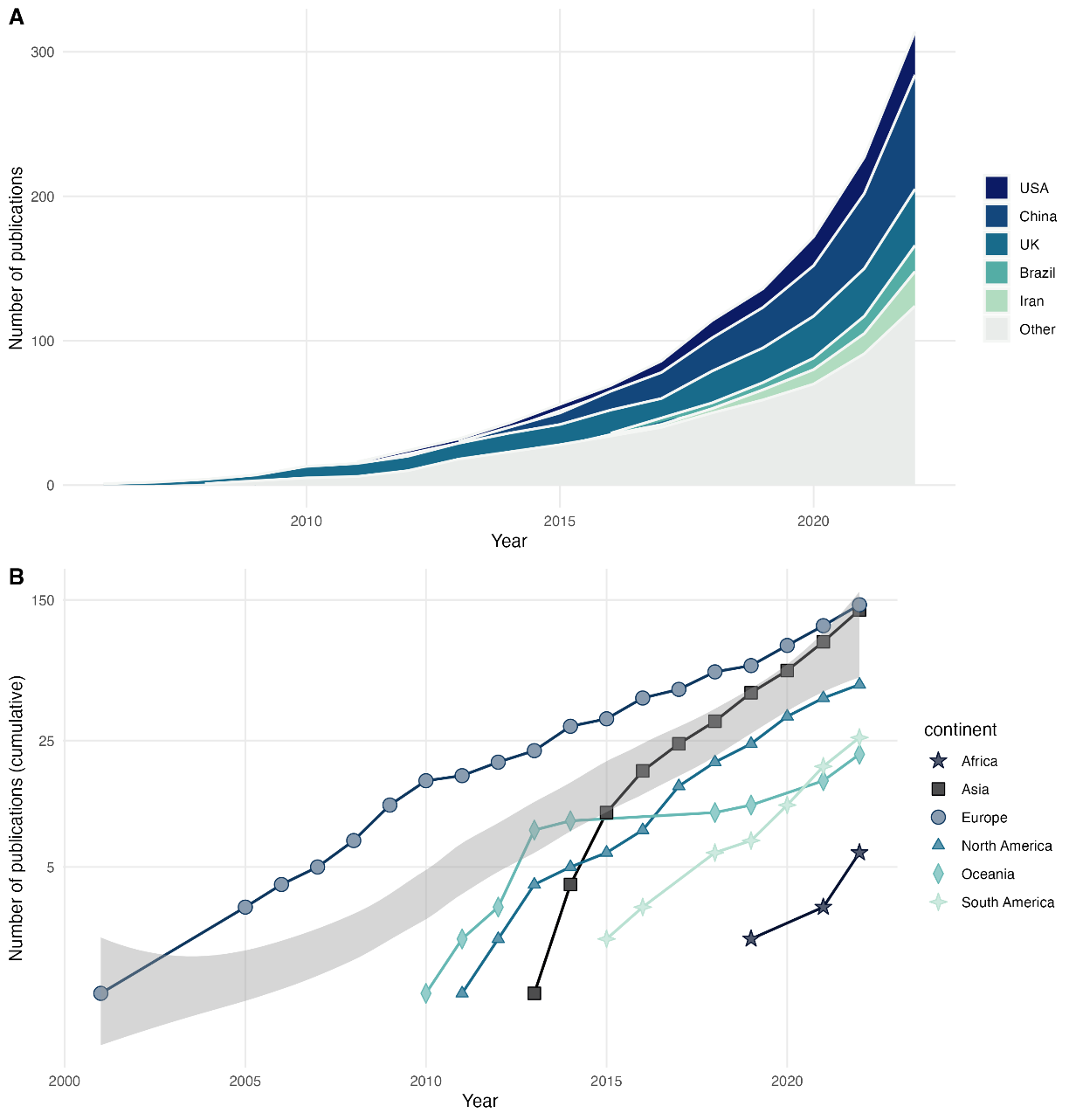


The 5 most prolific countries remain the USA, China, UK, Brazil, and Iran when only including systematic reviews without meta-analysis (A). Europe remained the most prolific continent in the production of systematic review (B). The median global growth of systematic reviews was 26%.

**Supplementary Figure 2**: Quality of animal systematic reviews (not including meta-analyses).


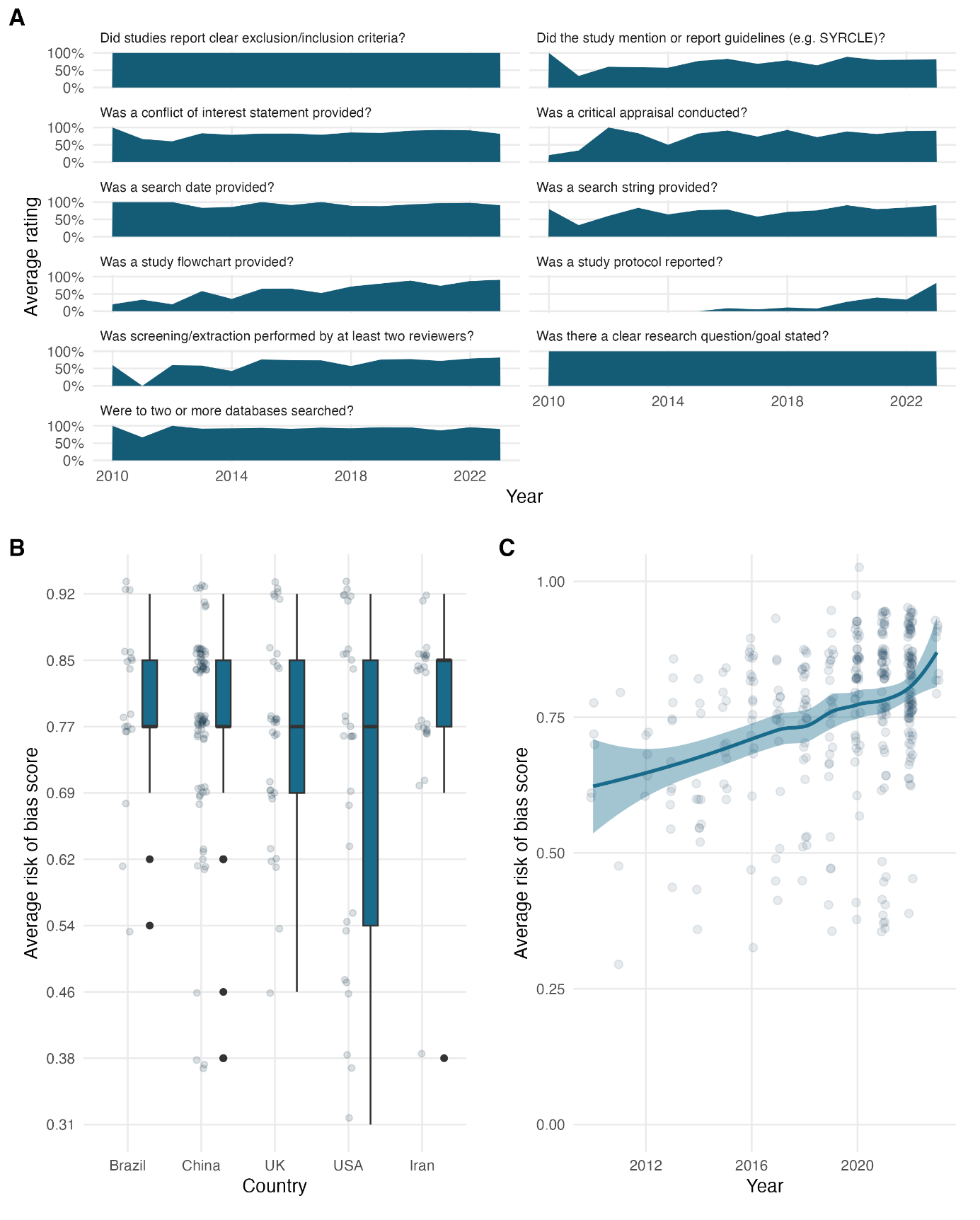


Quality of animal systematic reviews overall (A), per scored item (B), and for the most prolific countries publishing animal systematic reviews (C). There is a significant increase in systematic review quality over time. The countries show no statistically significant quality differences.

**Supplementary Table 1: Systematic review growth rates**

| Country or Group | **1993** | **1994** | **1995** | **1996** | **1997** | **1998** | **1999** | **2000** | **2001** | **2002** | **2003** | **2004** | **2005** | **2006** | **2007** | **2008** | **2009** | **2010** | **2011** | **2012** | **2013** | **2014** | **2015** | **2016** | **2017** | **2018** | **2019** | **2020** | **2021** | **2022** | **2023** |
| --- | --- | --- | --- | --- | --- | --- | --- | --- | --- | --- | --- | --- | --- | --- | --- | --- | --- | --- | --- | --- | --- | --- | --- | --- | --- | --- | --- | --- | --- | --- | --- |
| USA | 0 | 0 | 0 | 0 | 1 (+ 1 ) | 1 (+ 0 ) | 1 (+ 0 ) | 3 (+ 2 , + 200 % ) | 5 (+ 2 , + 67 % ) | 5 (+ 0 ) | 7 (+ 2 , + 40 % ) | 7 (+ 0 ) | 9 (+ 2 , + 29 % ) | 10 (+ 1 , + 11 % ) | 11 (+ 1 , + 10 % ) | 12 (+ 1 , + 9 % ) | 16 (+ 4 , + 33 % ) | 19 (+ 3 , + 19 % ) | 22 (+ 3 , + 16 % ) | 29 (+ 7 , + 32 % ) | 31 (+ 2 , + 7 % ) | 42 (+ 11 , + 35 % ) | 49 (+ 7 , + 17 % ) | 60 (+ 11 , + 22 % ) | 73 (+ 13 , + 22 % ) | 90 (+ 17 , + 23 % ) | 113 (+ 23 , + 26 % ) | 138 (+ 25 , + 22 % ) | 178 (+ 40 , + 29 % ) | 215 (+ 37 , + 21 % ) | 216 (+ 1 , + 0 % ) |
| China | 0 | 0 | 0 | 0 | 0 (+ 0 ) | 0 (+ 0 ) | 0 (+ 0 ) | 0 (+ 0 ) | 0 (+ 0 ) | 0 (+ 0 ) | 0 (+ 0 ) | 0 (+ 0 ) | 0 (+ 0 ) | 0 (+ 0 ) | 0 (+ 0 ) | 0 (+ 0 ) | 0 (+ 0 ) | 0 (+ 0 ) | 0 (+ 0 ) | 1 (+ 1 ) | 4 (+ 3 , + 300 % ) | 10 (+ 6 , + 150 % ) | 16 (+ 6 , + 60 % ) | 25 (+ 9 , + 56 % ) | 36 (+ 11 , + 44 % ) | 46 (+ 10 , + 28 % ) | 62 (+ 16 , + 35 % ) | 86 (+ 24 , + 39 % ) | 117 (+ 31 , + 36 % ) | 175 (+ 58 , + 50 % ) | 182 (+ 7 , + 4 % ) |
| UK | 0 | 0 | 0 | 0 | 0 (+ 0 ) | 0 (+ 0 ) | 0 (+ 0 ) | 1 (+ 1 ) | 1 (+ 0 ) | 1 (+ 0 ) | 1 (+ 0 ) | 3 (+ 2 , + 200 % ) | 9 (+ 6 , + 200 % ) | 11 (+ 2 , + 22 % ) | 13 (+ 2 , + 18 % ) | 18 (+ 5 , + 38 % ) | 25 (+ 7 , + 39 % ) | 32 (+ 7 , + 28 % ) | 38 (+ 6 , + 19 % ) | 41 (+ 3 , + 8 % ) | 45 (+ 4 , + 10 % ) | 52 (+ 7 , + 16 % ) | 56 (+ 4 , + 8 % ) | 63 (+ 7 , + 12 % ) | 67 (+ 4 , + 6 % ) | 77 (+ 10 , + 15 % ) | 84 (+ 7 , + 9 % ) | 98 (+ 14 , + 17 % ) | 120 (+ 22 , + 22 % ) | 142 (+ 22 , + 18 % ) | 144 (+ 2 , + 1 % ) |
| Brazil | 0 | 0 | 0 | 0 | 0 (+ 0 ) | 0 (+ 0 ) | 0 (+ 0 ) | 0 (+ 0 ) | 0 (+ 0 ) | 0 (+ 0 ) | 0 (+ 0 ) | 0 (+ 0 ) | 0 (+ 0 ) | 0 (+ 0 ) | 0 (+ 0 ) | 0 (+ 0 ) | 0 (+ 0 ) | 0 (+ 0 ) | 2 (+ 2 ) | 3 (+ 1 , + 50 % ) | 4 (+ 1 , + 33 % ) | 7 (+ 3 , + 75 % ) | 12 (+ 5 , + 71 % ) | 16 (+ 4 , + 33 % ) | 20 (+ 4 , + 25 % ) | 28 (+ 8 , + 40 % ) | 35 (+ 7 , + 25 % ) | 41 (+ 6 , + 17 % ) | 61 (+ 20 , + 49 % ) | 87 (+ 26 , + 43 % ) | 89 (+ 2 , + 2 % ) |
| Iran | 0 | 0 | 0 | 0 | 0 (+ 0 ) | 0 (+ 0 ) | 0 (+ 0 ) | 0 (+ 0 ) | 0 (+ 0 ) | 0 (+ 0 ) | 0 (+ 0 ) | 0 (+ 0 ) | 0 (+ 0 ) | 0 (+ 0 ) | 0 (+ 0 ) | 0 (+ 0 ) | 0 (+ 0 ) | 0 (+ 0 ) | 0 (+ 0 ) | 0 (+ 0 ) | 0 (+ 0 ) | 0 (+ 0 ) | 1 (+ 1 ) | 3 (+ 2 , + 200 % ) | 7 (+ 4 , + 133 % ) | 10 (+ 3 , + 43 % ) | 20 (+ 10 , + 100 % ) | 38 (+ 18 , + 90 % ) | 57 (+ 19 , + 50 % ) | 85 (+ 28 , + 49 % ) | 87 (+ 2 , + 2 % ) |
| Canada | 0 | 0 | 0 | 0 | 0 (+ 0 ) | 0 (+ 0 ) | 0 (+ 0 ) | 0 (+ 0 ) | 0 (+ 0 ) | 0 (+ 0 ) | 0 (+ 0 ) | 0 (+ 0 ) | 0 (+ 0 ) | 1 (+ 1 ) | 1 (+ 0 ) | 3 (+ 2 , + 200 % ) | 4 (+ 1 , + 33 % ) | 6 (+ 2 , + 50 % ) | 13 (+ 7 , + 117 % ) | 15 (+ 2 , + 15 % ) | 18 (+ 3 , + 20 % ) | 22 (+ 4 , + 22 % ) | 25 (+ 3 , + 14 % ) | 30 (+ 5 , + 20 % ) | 35 (+ 5 , + 17 % ) | 40 (+ 5 , + 14 % ) | 48 (+ 8 , + 20 % ) | 56 (+ 8 , + 17 % ) | 67 (+ 11 , + 20 % ) | 78 (+ 11 , + 16 % ) | 78 (+ 0 ) |
| Netherlands | 0 | 0 | 0 | 0 | 0 (+ 0 ) | 0 (+ 0 ) | 0 (+ 0 ) | 0 (+ 0 ) | 2 (+ 2 ) | 2 (+ 0 ) | 3 (+ 1 , + 50 % ) | 3 (+ 0 ) | 3 (+ 0 ) | 3 (+ 0 ) | 4 (+ 1 , + 33 % ) | 4 (+ 0 ) | 5 (+ 1 , + 25 % ) | 5 (+ 0 ) | 5 (+ 0 ) | 7 (+ 2 , + 40 % ) | 9 (+ 2 , + 29 % ) | 11 (+ 2 , + 22 % ) | 14 (+ 3 , + 27 % ) | 19 (+ 5 , + 36 % ) | 22 (+ 3 , + 16 % ) | 30 (+ 8 , + 36 % ) | 38 (+ 8 , + 27 % ) | 40 (+ 2 , + 5 % ) | 49 (+ 9 , + 22 % ) | 59 (+ 10 , + 20 % ) | 62 (+ 3 , + 5 % ) |
| Australia | 0 | 0 | 0 | 0 | 0 (+ 0 ) | 0 (+ 0 ) | 0 (+ 0 ) | 0 (+ 0 ) | 0 (+ 0 ) | 0 (+ 0 ) | 0 (+ 0 ) | 0 (+ 0 ) | 0 (+ 0 ) | 0 (+ 0 ) | 0 (+ 0 ) | 0 (+ 0 ) | 0 (+ 0 ) | 1 (+ 1 ) | 5 (+ 4 , + 400 % ) | 8 (+ 3 , + 60 % ) | 15 (+ 7 , + 88 % ) | 16 (+ 1 , + 7 % ) | 17 (+ 1 , + 6 % ) | 20 (+ 3 , + 18 % ) | 22 (+ 2 , + 10 % ) | 24 (+ 2 , + 9 % ) | 31 (+ 7 , + 29 % ) | 35 (+ 4 , + 13 % ) | 46 (+ 11 , + 31 % ) | 60 (+ 14 , + 30 % ) | 61 (+ 1 , + 2 % ) |
| Italy | 0 | 0 | 0 | 0 | 0 (+ 0 ) | 0 (+ 0 ) | 0 (+ 0 ) | 0 (+ 0 ) | 0 (+ 0 ) | 0 (+ 0 ) | 1 (+ 1 ) | 1 (+ 0 ) | 1 (+ 0 ) | 1 (+ 0 ) | 1 (+ 0 ) | 2 (+ 1 , + 100 % ) | 2 (+ 0 ) | 3 (+ 1 , + 50 % ) | 3 (+ 0 ) | 4 (+ 1 , + 33 % ) | 7 (+ 3 , + 75 % ) | 7 (+ 0 ) | 10 (+ 3 , + 43 % ) | 12 (+ 2 , + 20 % ) | 15 (+ 3 , + 25 % ) | 18 (+ 3 , + 20 % ) | 23 (+ 5 , + 28 % ) | 31 (+ 8 , + 35 % ) | 42 (+ 11 , + 35 % ) | 60 (+ 18 , + 43 % ) | 61 (+ 1 , + 2 % ) |
| Germany | 0 | 0 | 0 | 0 | 0 (+ 0 ) | 0 (+ 0 ) | 0 (+ 0 ) | 0 (+ 0 ) | 0 (+ 0 ) | 0 (+ 0 ) | 1 (+ 1 ) | 1 (+ 0 ) | 1 (+ 0 ) | 1 (+ 0 ) | 1 (+ 0 ) | 3 (+ 2 , + 200 % ) | 4 (+ 1 , + 33 % ) | 5 (+ 1 , + 25 % ) | 7 (+ 2 , + 40 % ) | 8 (+ 1 , + 14 % ) | 14 (+ 6 , + 75 % ) | 18 (+ 4 , + 29 % ) | 19 (+ 1 , + 6 % ) | 25 (+ 6 , + 32 % ) | 29 (+ 4 , + 16 % ) | 32 (+ 3 , + 10 % ) | 37 (+ 5 , + 16 % ) | 42 (+ 5 , + 14 % ) | 52 (+ 10 , + 24 % ) | 60 (+ 8 , + 15 % ) | 60 (+ 0 ) |
| France | 0 | 0 | 0 | 0 | 0 (+ 0 ) | 0 (+ 0 ) | 0 (+ 0 ) | 0 (+ 0 ) | 0 (+ 0 ) | 0 (+ 0 ) | 0 (+ 0 ) | 0 (+ 0 ) | 0 (+ 0 ) | 1 (+ 1 ) | 1 (+ 0 ) | 1 (+ 0 ) | 1 (+ 0 ) | 2 (+ 1 , + 100 % ) | 2 (+ 0 ) | 3 (+ 1 , + 50 % ) | 3 (+ 0 ) | 6 (+ 3 , + 100 % ) | 7 (+ 1 , + 17 % ) | 10 (+ 3 , + 43 % ) | 11 (+ 1 , + 10 % ) | 13 (+ 2 , + 18 % ) | 17 (+ 4 , + 31 % ) | 21 (+ 4 , + 24 % ) | 29 (+ 8 , + 38 % ) | 33 (+ 4 , + 14 % ) | 33 (+ 0 ) |
| Denmark | 0 | 0 | 0 | 0 | 0 (+ 0 ) | 0 (+ 0 ) | 0 (+ 0 ) | 0 (+ 0 ) | 0 (+ 0 ) | 0 (+ 0 ) | 0 (+ 0 ) | 0 (+ 0 ) | 0 (+ 0 ) | 0 (+ 0 ) | 0 (+ 0 ) | 0 (+ 0 ) | 0 (+ 0 ) | 0 (+ 0 ) | 0 (+ 0 ) | 1 (+ 1 ) | 1 (+ 0 ) | 5 (+ 4 , + 400 % ) | 6 (+ 1 , + 20 % ) | 8 (+ 2 , + 33 % ) | 11 (+ 3 , + 38 % ) | 15 (+ 4 , + 36 % ) | 18 (+ 3 , + 20 % ) | 18 (+ 0 ) | 23 (+ 5 , + 28 % ) | 31 (+ 8 , + 35 % ) | 31 (+ 0 ) |
| Spain | 0 | 0 | 0 | 0 | 0 (+ 0 ) | 0 (+ 0 ) | 0 (+ 0 ) | 0 (+ 0 ) | 0 (+ 0 ) | 0 (+ 0 ) | 0 (+ 0 ) | 0 (+ 0 ) | 0 (+ 0 ) | 0 (+ 0 ) | 0 (+ 0 ) | 1 (+ 1 ) | 1 (+ 0 ) | 1 (+ 0 ) | 1 (+ 0 ) | 3 (+ 2 , + 200 % ) | 3 (+ 0 ) | 4 (+ 1 , + 33 % ) | 4 (+ 0 ) | 5 (+ 1 , + 25 % ) | 6 (+ 1 , + 20 % ) | 7 (+ 1 , + 17 % ) | 7 (+ 0 ) | 13 (+ 6 , + 86 % ) | 21 (+ 8 , + 62 % ) | 31 (+ 10 , + 48 % ) | 31 (+ 0 ) |
| India | 0 | 0 | 0 | 0 | 0 (+ 0 ) | 0 (+ 0 ) | 0 (+ 0 ) | 0 (+ 0 ) | 0 (+ 0 ) | 0 (+ 0 ) | 0 (+ 0 ) | 0 (+ 0 ) | 0 (+ 0 ) | 0 (+ 0 ) | 0 (+ 0 ) | 0 (+ 0 ) | 0 (+ 0 ) | 0 (+ 0 ) | 0 (+ 0 ) | 0 (+ 0 ) | 0 (+ 0 ) | 0 (+ 0 ) | 1 (+ 1 ) | 3 (+ 2 , + 200 % ) | 4 (+ 1 , + 33 % ) | 7 (+ 3 , + 75 % ) | 8 (+ 1 , + 14 % ) | 11 (+ 3 , + 38 % ) | 17 (+ 6 , + 55 % ) | 21 (+ 4 , + 24 % ) | 23 (+ 2 , + 10 % ) |
| Malaysia | 0 | 0 | 0 | 0 | 0 (+ 0 ) | 0 (+ 0 ) | 0 (+ 0 ) | 0 (+ 0 ) | 0 (+ 0 ) | 0 (+ 0 ) | 0 (+ 0 ) | 0 (+ 0 ) | 0 (+ 0 ) | 0 (+ 0 ) | 0 (+ 0 ) | 0 (+ 0 ) | 0 (+ 0 ) | 1 (+ 1 ) | 1 (+ 0 ) | 1 (+ 0 ) | 1 (+ 0 ) | 1 (+ 0 ) | 1 (+ 0 ) | 1 (+ 0 ) | 2 (+ 1 , + 100 % ) | 3 (+ 1 , + 50 % ) | 4 (+ 1 , + 33 % ) | 7 (+ 3 , + 75 % ) | 12 (+ 5 , + 71 % ) | 18 (+ 6 , + 50 % ) | 19 (+ 1 , + 6 % ) |
| Switzerland | 0 | 0 | 0 | 0 | 0 (+ 0 ) | 0 (+ 0 ) | 0 (+ 0 ) | 0 (+ 0 ) | 0 (+ 0 ) | 0 (+ 0 ) | 0 (+ 0 ) | 0 (+ 0 ) | 0 (+ 0 ) | 0 (+ 0 ) | 0 (+ 0 ) | 0 (+ 0 ) | 0 (+ 0 ) | 0 (+ 0 ) | 1 (+ 1 ) | 1 (+ 0 ) | 1 (+ 0 ) | 2 (+ 1 , + 100 % ) | 3 (+ 1 , + 50 % ) | 3 (+ 0 ) | 4 (+ 1 , + 33 % ) | 5 (+ 1 , + 25 % ) | 9 (+ 4 , + 80 % ) | 12 (+ 3 , + 33 % ) | 13 (+ 1 , + 8 % ) | 17 (+ 4 , + 31 % ) | 17 (+ 0 ) |
| Sweden | 0 | 0 | 0 | 0 | 0 (+ 0 ) | 0 (+ 0 ) | 0 (+ 0 ) | 0 (+ 0 ) | 0 (+ 0 ) | 0 (+ 0 ) | 0 (+ 0 ) | 0 (+ 0 ) | 0 (+ 0 ) | 0 (+ 0 ) | 0 (+ 0 ) | 0 (+ 0 ) | 0 (+ 0 ) | 1 (+ 1 ) | 2 (+ 1 , + 100 % ) | 2 (+ 0 ) | 3 (+ 1 , + 50 % ) | 4 (+ 1 , + 33 % ) | 5 (+ 1 , + 25 % ) | 6 (+ 1 , + 20 % ) | 6 (+ 0 ) | 6 (+ 0 ) | 6 (+ 0 ) | 8 (+ 2 , + 33 % ) | 12 (+ 4 , + 50 % ) | 15 (+ 3 , + 25 % ) | 15 (+ 0 ) |
| Belgium | 0 | 0 | 0 | 0 | 0 (+ 0 ) | 0 (+ 0 ) | 0 (+ 0 ) | 0 (+ 0 ) | 0 (+ 0 ) | 0 (+ 0 ) | 1 (+ 1 ) | 1 (+ 0 ) | 1 (+ 0 ) | 1 (+ 0 ) | 1 (+ 0 ) | 1 (+ 0 ) | 1 (+ 0 ) | 1 (+ 0 ) | 1 (+ 0 ) | 1 (+ 0 ) | 1 (+ 0 ) | 1 (+ 0 ) | 1 (+ 0 ) | 1 (+ 0 ) | 1 (+ 0 ) | 4 (+ 3 , + 300 % ) | 4 (+ 0 ) | 5 (+ 1 , + 25 % ) | 10 (+ 5 , + 100 % ) | 13 (+ 3 , + 30 % ) | 13 (+ 0 ) |
| Portugal | 0 | 0 | 0 | 0 | 0 (+ 0 ) | 0 (+ 0 ) | 0 (+ 0 ) | 0 (+ 0 ) | 0 (+ 0 ) | 0 (+ 0 ) | 0 (+ 0 ) | 0 (+ 0 ) | 0 (+ 0 ) | 0 (+ 0 ) | 0 (+ 0 ) | 0 (+ 0 ) | 0 (+ 0 ) | 0 (+ 0 ) | 0 (+ 0 ) | 0 (+ 0 ) | 0 (+ 0 ) | 0 (+ 0 ) | 0 (+ 0 ) | 1 (+ 1 ) | 1 (+ 0 ) | 1 (+ 0 ) | 2 (+ 1 , + 100 % ) | 4 (+ 2 , + 100 % ) | 8 (+ 4 , + 100 % ) | 11 (+ 3 , + 38 % ) | 11 (+ 0 ) |
| South Korea | 0 | 0 | 0 | 0 | 0 (+ 0 ) | 0 (+ 0 ) | 0 (+ 0 ) | 0 (+ 0 ) | 0 (+ 0 ) | 0 (+ 0 ) | 0 (+ 0 ) | 0 (+ 0 ) | 0 (+ 0 ) | 0 (+ 0 ) | 0 (+ 0 ) | 0 (+ 0 ) | 0 (+ 0 ) | 0 (+ 0 ) | 0 (+ 0 ) | 0 (+ 0 ) | 0 (+ 0 ) | 0 (+ 0 ) | 0 (+ 0 ) | 1 (+ 1 ) | 2 (+ 1 , + 100 % ) | 3 (+ 1 , + 50 % ) | 6 (+ 3 , + 100 % ) | 6 (+ 0 ) | 9 (+ 3 , + 50 % ) | 11 (+ 2 , + 22 % ) | 11 (+ 0 ) |
| Greece | 0 | 0 | 0 | 0 | 0 (+ 0 ) | 0 (+ 0 ) | 0 (+ 0 ) | 0 (+ 0 ) | 0 (+ 0 ) | 0 (+ 0 ) | 0 (+ 0 ) | 0 (+ 0 ) | 0 (+ 0 ) | 0 (+ 0 ) | 0 (+ 0 ) | 1 (+ 1 ) | 1 (+ 0 ) | 3 (+ 2 , + 200 % ) | 3 (+ 0 ) | 4 (+ 1 , + 33 % ) | 4 (+ 0 ) | 4 (+ 0 ) | 4 (+ 0 ) | 4 (+ 0 ) | 4 (+ 0 ) | 5 (+ 1 , + 25 % ) | 5 (+ 0 ) | 5 (+ 0 ) | 7 (+ 2 , + 40 % ) | 9 (+ 2 , + 29 % ) | 9 (+ 0 ) |
| Poland | 0 | 0 | 0 | 0 | 0 (+ 0 ) | 0 (+ 0 ) | 0 (+ 0 ) | 0 (+ 0 ) | 0 (+ 0 ) | 0 (+ 0 ) | 0 (+ 0 ) | 0 (+ 0 ) | 0 (+ 0 ) | 0 (+ 0 ) | 0 (+ 0 ) | 0 (+ 0 ) | 1 (+ 1 ) | 2 (+ 1 , + 100 % ) | 2 (+ 0 ) | 2 (+ 0 ) | 2 (+ 0 ) | 2 (+ 0 ) | 2 (+ 0 ) | 2 (+ 0 ) | 2 (+ 0 ) | 3 (+ 1 , + 50 % ) | 4 (+ 1 , + 33 % ) | 7 (+ 3 , + 75 % ) | 8 (+ 1 , + 14 % ) | 9 (+ 1 , + 12 % ) | 9 (+ 0 ) |
| Austria | 0 | 0 | 0 | 0 | 0 (+ 0 ) | 0 (+ 0 ) | 0 (+ 0 ) | 0 (+ 0 ) | 0 (+ 0 ) | 0 (+ 0 ) | 0 (+ 0 ) | 0 (+ 0 ) | 1 (+ 1 ) | 1 (+ 0 ) | 1 (+ 0 ) | 1 (+ 0 ) | 1 (+ 0 ) | 1 (+ 0 ) | 1 (+ 0 ) | 1 (+ 0 ) | 1 (+ 0 ) | 2 (+ 1 , + 100 % ) | 3 (+ 1 , + 50 % ) | 3 (+ 0 ) | 4 (+ 1 , + 33 % ) | 4 (+ 0 ) | 5 (+ 1 , + 25 % ) | 7 (+ 2 , + 40 % ) | 7 (+ 0 ) | 8 (+ 1 , + 14 % ) | 8 (+ 0 ) |
| Mexico | 0 | 0 | 0 | 0 | 0 (+ 0 ) | 0 (+ 0 ) | 0 (+ 0 ) | 1 (+ 1 ) | 1 (+ 0 ) | 1 (+ 0 ) | 1 (+ 0 ) | 1 (+ 0 ) | 1 (+ 0 ) | 1 (+ 0 ) | 1 (+ 0 ) | 1 (+ 0 ) | 1 (+ 0 ) | 1 (+ 0 ) | 1 (+ 0 ) | 1 (+ 0 ) | 1 (+ 0 ) | 1 (+ 0 ) | 3 (+ 2 , + 200 % ) | 3 (+ 0 ) | 3 (+ 0 ) | 3 (+ 0 ) | 3 (+ 0 ) | 3 (+ 0 ) | 4 (+ 1 , + 33 % ) | 8 (+ 4 , + 100 % ) | 8 (+ 0 ) |
| New Zealand | 0 | 0 | 0 | 0 | 0 (+ 0 ) | 0 (+ 0 ) | 0 (+ 0 ) | 0 (+ 0 ) | 0 (+ 0 ) | 0 (+ 0 ) | 0 (+ 0 ) | 0 (+ 0 ) | 0 (+ 0 ) | 0 (+ 0 ) | 0 (+ 0 ) | 0 (+ 0 ) | 0 (+ 0 ) | 0 (+ 0 ) | 0 (+ 0 ) | 1 (+ 1 ) | 1 (+ 0 ) | 1 (+ 0 ) | 1 (+ 0 ) | 1 (+ 0 ) | 1 (+ 0 ) | 1 (+ 0 ) | 1 (+ 0 ) | 2 (+ 1 , + 100 % ) | 5 (+ 3 , + 150 % ) | 8 (+ 3 , + 60 % ) | 8 (+ 0 ) |
| Russia | 0 | 0 | 0 | 0 | 0 (+ 0 ) | 0 (+ 0 ) | 0 (+ 0 ) | 0 (+ 0 ) | 0 (+ 0 ) | 0 (+ 0 ) | 0 (+ 0 ) | 0 (+ 0 ) | 0 (+ 0 ) | 0 (+ 0 ) | 0 (+ 0 ) | 0 (+ 0 ) | 0 (+ 0 ) | 0 (+ 0 ) | 0 (+ 0 ) | 0 (+ 0 ) | 0 (+ 0 ) | 0 (+ 0 ) | 0 (+ 0 ) | 0 (+ 0 ) | 0 (+ 0 ) | 0 (+ 0 ) | 1 (+ 1 ) | 2 (+ 1 , + 100 % ) | 3 (+ 1 , + 50 % ) | 8 (+ 5 , + 167 % ) | 8 (+ 0 ) |
| Colombia | 0 | 0 | 0 | 0 | 0 (+ 0 ) | 0 (+ 0 ) | 0 (+ 0 ) | 0 (+ 0 ) | 0 (+ 0 ) | 0 (+ 0 ) | 0 (+ 0 ) | 0 (+ 0 ) | 0 (+ 0 ) | 0 (+ 0 ) | 1 (+ 1 ) | 1 (+ 0 ) | 1 (+ 0 ) | 1 (+ 0 ) | 1 (+ 0 ) | 1 (+ 0 ) | 1 (+ 0 ) | 1 (+ 0 ) | 1 (+ 0 ) | 1 (+ 0 ) | 2 (+ 1 , + 100 % ) | 3 (+ 1 , + 50 % ) | 3 (+ 0 ) | 3 (+ 0 ) | 4 (+ 1 , + 33 % ) | 7 (+ 3 , + 75 % ) | 7 (+ 0 ) |
| Ireland | 0 | 0 | 0 | 0 | 0 (+ 0 ) | 0 (+ 0 ) | 0 (+ 0 ) | 0 (+ 0 ) | 0 (+ 0 ) | 0 (+ 0 ) | 0 (+ 0 ) | 0 (+ 0 ) | 0 (+ 0 ) | 0 (+ 0 ) | 0 (+ 0 ) | 0 (+ 0 ) | 0 (+ 0 ) | 0 (+ 0 ) | 0 (+ 0 ) | 0 (+ 0 ) | 0 (+ 0 ) | 0 (+ 0 ) | 0 (+ 0 ) | 1 (+ 1 ) | 1 (+ 0 ) | 1 (+ 0 ) | 2 (+ 1 , + 100 % ) | 3 (+ 1 , + 50 % ) | 6 (+ 3 , + 100 % ) | 7 (+ 1 , + 17 % ) | 7 (+ 0 ) |
| Japan | 0 | 0 | 0 | 0 | 0 (+ 0 ) | 0 (+ 0 ) | 0 (+ 0 ) | 0 (+ 0 ) | 0 (+ 0 ) | 0 (+ 0 ) | 0 (+ 0 ) | 0 (+ 0 ) | 0 (+ 0 ) | 0 (+ 0 ) | 0 (+ 0 ) | 0 (+ 0 ) | 0 (+ 0 ) | 0 (+ 0 ) | 0 (+ 0 ) | 1 (+ 1 ) | 1 (+ 0 ) | 1 (+ 0 ) | 1 (+ 0 ) | 1 (+ 0 ) | 1 (+ 0 ) | 3 (+ 2 , + 200 % ) | 3 (+ 0 ) | 6 (+ 3 , + 100 % ) | 7 (+ 1 , + 17 % ) | 7 (+ 0 ) | 7 (+ 0 ) |
| Georgia | 0 | 0 | 0 | 0 | 0 (+ 0 ) | 0 (+ 0 ) | 0 (+ 0 ) | 0 (+ 0 ) | 0 (+ 0 ) | 0 (+ 0 ) | 0 (+ 0 ) | 0 (+ 0 ) | 0 (+ 0 ) | 0 (+ 0 ) | 0 (+ 0 ) | 0 (+ 0 ) | 0 (+ 0 ) | 0 (+ 0 ) | 0 (+ 0 ) | 0 (+ 0 ) | 0 (+ 0 ) | 0 (+ 0 ) | 3 (+ 3 ) | 4 (+ 1 , + 33 % ) | 5 (+ 1 , + 25 % ) | 6 (+ 1 , + 20 % ) | 6 (+ 0 ) | 6 (+ 0 ) | 6 (+ 0 ) | 6 (+ 0 ) | 6 (+ 0 ) |
| Israel | 0 | 0 | 0 | 0 | 0 (+ 0 ) | 0 (+ 0 ) | 0 (+ 0 ) | 0 (+ 0 ) | 0 (+ 0 ) | 0 (+ 0 ) | 0 (+ 0 ) | 0 (+ 0 ) | 0 (+ 0 ) | 0 (+ 0 ) | 0 (+ 0 ) | 0 (+ 0 ) | 0 (+ 0 ) | 0 (+ 0 ) | 0 (+ 0 ) | 1 (+ 1 ) | 1 (+ 0 ) | 1 (+ 0 ) | 2 (+ 1 , + 100 % ) | 2 (+ 0 ) | 2 (+ 0 ) | 2 (+ 0 ) | 3 (+ 1 , + 50 % ) | 6 (+ 3 , + 100 % ) | 6 (+ 0 ) | 6 (+ 0 ) | 6 (+ 0 ) |
| Croatia | 0 | 0 | 0 | 0 | 0 (+ 0 ) | 0 (+ 0 ) | 0 (+ 0 ) | 0 (+ 0 ) | 0 (+ 0 ) | 0 (+ 0 ) | 0 (+ 0 ) | 0 (+ 0 ) | 0 (+ 0 ) | 0 (+ 0 ) | 0 (+ 0 ) | 0 (+ 0 ) | 0 (+ 0 ) | 0 (+ 0 ) | 0 (+ 0 ) | 0 (+ 0 ) | 0 (+ 0 ) | 0 (+ 0 ) | 0 (+ 0 ) | 0 (+ 0 ) | 0 (+ 0 ) | 1 (+ 1 ) | 1 (+ 0 ) | 2 (+ 1 , + 100 % ) | 2 (+ 0 ) | 4 (+ 2 , + 100 % ) | 4 (+ 0 ) |
| Egypt | 0 | 0 | 0 | 0 | 0 (+ 0 ) | 0 (+ 0 ) | 0 (+ 0 ) | 0 (+ 0 ) | 0 (+ 0 ) | 0 (+ 0 ) | 0 (+ 0 ) | 0 (+ 0 ) | 0 (+ 0 ) | 0 (+ 0 ) | 0 (+ 0 ) | 0 (+ 0 ) | 0 (+ 0 ) | 0 (+ 0 ) | 0 (+ 0 ) | 0 (+ 0 ) | 0 (+ 0 ) | 0 (+ 0 ) | 0 (+ 0 ) | 0 (+ 0 ) | 0 (+ 0 ) | 0 (+ 0 ) | 1 (+ 1 ) | 1 (+ 0 ) | 3 (+ 2 , + 200 % ) | 4 (+ 1 , + 33 % ) | 4 (+ 0 ) |
| Indonesia | 0 | 0 | 0 | 0 | 0 (+ 0 ) | 0 (+ 0 ) | 0 (+ 0 ) | 0 (+ 0 ) | 0 (+ 0 ) | 0 (+ 0 ) | 0 (+ 0 ) | 0 (+ 0 ) | 0 (+ 0 ) | 0 (+ 0 ) | 0 (+ 0 ) | 0 (+ 0 ) | 0 (+ 0 ) | 0 (+ 0 ) | 0 (+ 0 ) | 0 (+ 0 ) | 0 (+ 0 ) | 0 (+ 0 ) | 0 (+ 0 ) | 0 (+ 0 ) | 0 (+ 0 ) | 0 (+ 0 ) | 0 (+ 0 ) | 0 (+ 0 ) | 3 (+ 3 ) | 4 (+ 1 , + 33 % ) | 4 (+ 0 ) |
| Nigeria | 0 | 0 | 0 | 0 | 0 (+ 0 ) | 0 (+ 0 ) | 0 (+ 0 ) | 0 (+ 0 ) | 0 (+ 0 ) | 0 (+ 0 ) | 0 (+ 0 ) | 0 (+ 0 ) | 0 (+ 0 ) | 0 (+ 0 ) | 0 (+ 0 ) | 0 (+ 0 ) | 0 (+ 0 ) | 0 (+ 0 ) | 0 (+ 0 ) | 0 (+ 0 ) | 0 (+ 0 ) | 0 (+ 0 ) | 0 (+ 0 ) | 0 (+ 0 ) | 0 (+ 0 ) | 0 (+ 0 ) | 0 (+ 0 ) | 0 (+ 0 ) | 0 (+ 0 ) | 4 (+ 4 ) | 4 (+ 0 ) |
| Norway | 0 | 0 | 0 | 0 | 0 (+ 0 ) | 0 (+ 0 ) | 0 (+ 0 ) | 0 (+ 0 ) | 0 (+ 0 ) | 0 (+ 0 ) | 1 (+ 1 ) | 1 (+ 0 ) | 1 (+ 0 ) | 1 (+ 0 ) | 1 (+ 0 ) | 1 (+ 0 ) | 1 (+ 0 ) | 1 (+ 0 ) | 1 (+ 0 ) | 1 (+ 0 ) | 2 (+ 1 , + 100 % ) | 2 (+ 0 ) | 2 (+ 0 ) | 2 (+ 0 ) | 2 (+ 0 ) | 2 (+ 0 ) | 2 (+ 0 ) | 3 (+ 1 , + 50 % ) | 4 (+ 1 , + 33 % ) | 4 (+ 0 ) | 4 (+ 0 ) |
| Turkey | 0 | 0 | 0 | 0 | 0 (+ 0 ) | 0 (+ 0 ) | 0 (+ 0 ) | 0 (+ 0 ) | 0 (+ 0 ) | 0 (+ 0 ) | 0 (+ 0 ) | 0 (+ 0 ) | 0 (+ 0 ) | 0 (+ 0 ) | 0 (+ 0 ) | 0 (+ 0 ) | 0 (+ 0 ) | 0 (+ 0 ) | 0 (+ 0 ) | 0 (+ 0 ) | 0 (+ 0 ) | 0 (+ 0 ) | 0 (+ 0 ) | 0 (+ 0 ) | 0 (+ 0 ) | 0 (+ 0 ) | 1 (+ 1 ) | 1 (+ 0 ) | 1 (+ 0 ) | 4 (+ 3 , + 300 % ) | 4 (+ 0 ) |
| Romania | 0 | 0 | 0 | 0 | 0 (+ 0 ) | 0 (+ 0 ) | 0 (+ 0 ) | 0 (+ 0 ) | 0 (+ 0 ) | 0 (+ 0 ) | 0 (+ 0 ) | 0 (+ 0 ) | 0 (+ 0 ) | 0 (+ 0 ) | 0 (+ 0 ) | 0 (+ 0 ) | 0 (+ 0 ) | 0 (+ 0 ) | 0 (+ 0 ) | 0 (+ 0 ) | 0 (+ 0 ) | 0 (+ 0 ) | 0 (+ 0 ) | 0 (+ 0 ) | 0 (+ 0 ) | 0 (+ 0 ) | 0 (+ 0 ) | 1 (+ 1 ) | 2 (+ 1 , + 100 % ) | 2 (+ 0 ) | 3 (+ 1 , + 50 % ) |
| Singapore | 0 | 0 | 0 | 0 | 0 (+ 0 ) | 0 (+ 0 ) | 0 (+ 0 ) | 0 (+ 0 ) | 0 (+ 0 ) | 0 (+ 0 ) | 0 (+ 0 ) | 0 (+ 0 ) | 0 (+ 0 ) | 0 (+ 0 ) | 0 (+ 0 ) | 0 (+ 0 ) | 0 (+ 0 ) | 0 (+ 0 ) | 0 (+ 0 ) | 0 (+ 0 ) | 0 (+ 0 ) | 0 (+ 0 ) | 0 (+ 0 ) | 0 (+ 0 ) | 0 (+ 0 ) | 1 (+ 1 ) | 1 (+ 0 ) | 1 (+ 0 ) | 3 (+ 2 , + 200 % ) | 3 (+ 0 ) | 3 (+ 0 ) |
| South Africa | 0 | 0 | 0 | 0 | 0 (+ 0 ) | 0 (+ 0 ) | 0 (+ 0 ) | 0 (+ 0 ) | 0 (+ 0 ) | 0 (+ 0 ) | 0 (+ 0 ) | 0 (+ 0 ) | 0 (+ 0 ) | 0 (+ 0 ) | 0 (+ 0 ) | 0 (+ 0 ) | 0 (+ 0 ) | 0 (+ 0 ) | 0 (+ 0 ) | 0 (+ 0 ) | 0 (+ 0 ) | 0 (+ 0 ) | 0 (+ 0 ) | 0 (+ 0 ) | 0 (+ 0 ) | 0 (+ 0 ) | 1 (+ 1 ) | 1 (+ 0 ) | 2 (+ 1 , + 100 % ) | 3 (+ 1 , + 50 % ) | 3 (+ 0 ) |
| Taiwan | 0 | 0 | 0 | 0 | 0 (+ 0 ) | 0 (+ 0 ) | 0 (+ 0 ) | 0 (+ 0 ) | 0 (+ 0 ) | 0 (+ 0 ) | 0 (+ 0 ) | 0 (+ 0 ) | 0 (+ 0 ) | 0 (+ 0 ) | 0 (+ 0 ) | 0 (+ 0 ) | 0 (+ 0 ) | 0 (+ 0 ) | 0 (+ 0 ) | 0 (+ 0 ) | 0 (+ 0 ) | 0 (+ 0 ) | 0 (+ 0 ) | 0 (+ 0 ) | 0 (+ 0 ) | 0 (+ 0 ) | 1 (+ 1 ) | 1 (+ 0 ) | 3 (+ 2 , + 200 % ) | 3 (+ 0 ) | 3 (+ 0 ) |
| United Arab Emirates | 0 | 0 | 0 | 0 | 0 (+ 0 ) | 0 (+ 0 ) | 0 (+ 0 ) | 0 (+ 0 ) | 0 (+ 0 ) | 0 (+ 0 ) | 0 (+ 0 ) | 0 (+ 0 ) | 0 (+ 0 ) | 1 (+ 1 ) | 1 (+ 0 ) | 1 (+ 0 ) | 1 (+ 0 ) | 1 (+ 0 ) | 1 (+ 0 ) | 1 (+ 0 ) | 1 (+ 0 ) | 1 (+ 0 ) | 1 (+ 0 ) | 1 (+ 0 ) | 1 (+ 0 ) | 1 (+ 0 ) | 1 (+ 0 ) | 1 (+ 0 ) | 2 (+ 1 , + 100 % ) | 3 (+ 1 , + 50 % ) | 3 (+ 0 ) |
| Albania | 0 | 0 | 0 | 0 | 0 (+ 0 ) | 0 (+ 0 ) | 0 (+ 0 ) | 0 (+ 0 ) | 0 (+ 0 ) | 0 (+ 0 ) | 0 (+ 0 ) | 0 (+ 0 ) | 0 (+ 0 ) | 0 (+ 0 ) | 0 (+ 0 ) | 0 (+ 0 ) | 0 (+ 0 ) | 0 (+ 0 ) | 0 (+ 0 ) | 0 (+ 0 ) | 0 (+ 0 ) | 0 (+ 0 ) | 0 (+ 0 ) | 0 (+ 0 ) | 0 (+ 0 ) | 1 (+ 1 ) | 1 (+ 0 ) | 1 (+ 0 ) | 1 (+ 0 ) | 2 (+ 1 , + 100 % ) | 2 (+ 0 ) |
| Chile | 0 | 0 | 0 | 0 | 0 (+ 0 ) | 0 (+ 0 ) | 0 (+ 0 ) | 0 (+ 0 ) | 0 (+ 0 ) | 0 (+ 0 ) | 0 (+ 0 ) | 0 (+ 0 ) | 0 (+ 0 ) | 0 (+ 0 ) | 0 (+ 0 ) | 0 (+ 0 ) | 0 (+ 0 ) | 0 (+ 0 ) | 0 (+ 0 ) | 0 (+ 0 ) | 0 (+ 0 ) | 0 (+ 0 ) | 0 (+ 0 ) | 0 (+ 0 ) | 0 (+ 0 ) | 0 (+ 0 ) | 0 (+ 0 ) | 0 (+ 0 ) | 0 (+ 0 ) | 1 (+ 1 ) | 2 (+ 1 , + 100 % ) |
| Hungary | 0 | 0 | 0 | 0 | 0 (+ 0 ) | 0 (+ 0 ) | 0 (+ 0 ) | 0 (+ 0 ) | 0 (+ 0 ) | 0 (+ 0 ) | 0 (+ 0 ) | 0 (+ 0 ) | 0 (+ 0 ) | 0 (+ 0 ) | 0 (+ 0 ) | 0 (+ 0 ) | 0 (+ 0 ) | 0 (+ 0 ) | 0 (+ 0 ) | 0 (+ 0 ) | 0 (+ 0 ) | 0 (+ 0 ) | 0 (+ 0 ) | 0 (+ 0 ) | 1 (+ 1 ) | 1 (+ 0 ) | 2 (+ 1 , + 100 % ) | 2 (+ 0 ) | 2 (+ 0 ) | 2 (+ 0 ) | 2 (+ 0 ) |
| Vietnam | 0 | 0 | 0 | 0 | 0 (+ 0 ) | 0 (+ 0 ) | 0 (+ 0 ) | 0 (+ 0 ) | 0 (+ 0 ) | 0 (+ 0 ) | 0 (+ 0 ) | 0 (+ 0 ) | 0 (+ 0 ) | 0 (+ 0 ) | 0 (+ 0 ) | 0 (+ 0 ) | 0 (+ 0 ) | 0 (+ 0 ) | 0 (+ 0 ) | 0 (+ 0 ) | 0 (+ 0 ) | 0 (+ 0 ) | 0 (+ 0 ) | 0 (+ 0 ) | 0 (+ 0 ) | 1 (+ 1 ) | 1 (+ 0 ) | 1 (+ 0 ) | 2 (+ 1 , + 100 % ) | 2 (+ 0 ) | 2 (+ 0 ) |
| Argentina | 0 | 0 | 0 | 0 | 0 (+ 0 ) | 0 (+ 0 ) | 0 (+ 0 ) | 0 (+ 0 ) | 0 (+ 0 ) | 0 (+ 0 ) | 0 (+ 0 ) | 0 (+ 0 ) | 0 (+ 0 ) | 0 (+ 0 ) | 0 (+ 0 ) | 0 (+ 0 ) | 0 (+ 0 ) | 0 (+ 0 ) | 0 (+ 0 ) | 0 (+ 0 ) | 0 (+ 0 ) | 0 (+ 0 ) | 0 (+ 0 ) | 0 (+ 0 ) | 0 (+ 0 ) | 0 (+ 0 ) | 0 (+ 0 ) | 0 (+ 0 ) | 1 (+ 1 ) | 1 (+ 0 ) | 1 (+ 0 ) |
| Czech Republic | 0 | 0 | 0 | 0 | 0 (+ 0 ) | 0 (+ 0 ) | 0 (+ 0 ) | 0 (+ 0 ) | 0 (+ 0 ) | 0 (+ 0 ) | 0 (+ 0 ) | 0 (+ 0 ) | 0 (+ 0 ) | 0 (+ 0 ) | 0 (+ 0 ) | 0 (+ 0 ) | 0 (+ 0 ) | 0 (+ 0 ) | 0 (+ 0 ) | 0 (+ 0 ) | 0 (+ 0 ) | 0 (+ 0 ) | 0 (+ 0 ) | 0 (+ 0 ) | 0 (+ 0 ) | 0 (+ 0 ) | 0 (+ 0 ) | 0 (+ 0 ) | 1 (+ 1 ) | 1 (+ 0 ) | 1 (+ 0 ) |
| Ethiopia | 0 | 0 | 0 | 0 | 0 (+ 0 ) | 0 (+ 0 ) | 0 (+ 0 ) | 0 (+ 0 ) | 0 (+ 0 ) | 0 (+ 0 ) | 0 (+ 0 ) | 0 (+ 0 ) | 0 (+ 0 ) | 0 (+ 0 ) | 0 (+ 0 ) | 0 (+ 0 ) | 0 (+ 0 ) | 0 (+ 0 ) | 0 (+ 0 ) | 0 (+ 0 ) | 0 (+ 0 ) | 0 (+ 0 ) | 0 (+ 0 ) | 0 (+ 0 ) | 0 (+ 0 ) | 0 (+ 0 ) | 1 (+ 1 ) | 1 (+ 0 ) | 1 (+ 0 ) | 1 (+ 0 ) | 1 (+ 0 ) |
| Finland | 0 | 0 | 0 | 0 | 0 (+ 0 ) | 0 (+ 0 ) | 0 (+ 0 ) | 0 (+ 0 ) | 0 (+ 0 ) | 0 (+ 0 ) | 0 (+ 0 ) | 0 (+ 0 ) | 0 (+ 0 ) | 0 (+ 0 ) | 0 (+ 0 ) | 0 (+ 0 ) | 0 (+ 0 ) | 0 (+ 0 ) | 0 (+ 0 ) | 0 (+ 0 ) | 0 (+ 0 ) | 0 (+ 0 ) | 0 (+ 0 ) | 0 (+ 0 ) | 0 (+ 0 ) | 0 (+ 0 ) | 0 (+ 0 ) | 0 (+ 0 ) | 0 (+ 0 ) | 1 (+ 1 ) | 1 (+ 0 ) |
| Guatemala | 0 | 0 | 0 | 0 | 0 (+ 0 ) | 0 (+ 0 ) | 0 (+ 0 ) | 0 (+ 0 ) | 0 (+ 0 ) | 0 (+ 0 ) | 0 (+ 0 ) | 0 (+ 0 ) | 0 (+ 0 ) | 0 (+ 0 ) | 0 (+ 0 ) | 0 (+ 0 ) | 0 (+ 0 ) | 0 (+ 0 ) | 0 (+ 0 ) | 0 (+ 0 ) | 0 (+ 0 ) | 0 (+ 0 ) | 0 (+ 0 ) | 0 (+ 0 ) | 0 (+ 0 ) | 0 (+ 0 ) | 0 (+ 0 ) | 0 (+ 0 ) | 0 (+ 0 ) | 1 (+ 1 ) | 1 (+ 0 ) |
| Iraq | 0 | 0 | 0 | 0 | 0 (+ 0 ) | 0 (+ 0 ) | 0 (+ 0 ) | 0 (+ 0 ) | 0 (+ 0 ) | 0 (+ 0 ) | 0 (+ 0 ) | 0 (+ 0 ) | 0 (+ 0 ) | 0 (+ 0 ) | 0 (+ 0 ) | 0 (+ 0 ) | 0 (+ 0 ) | 0 (+ 0 ) | 0 (+ 0 ) | 0 (+ 0 ) | 0 (+ 0 ) | 0 (+ 0 ) | 0 (+ 0 ) | 0 (+ 0 ) | 0 (+ 0 ) | 0 (+ 0 ) | 0 (+ 0 ) | 0 (+ 0 ) | 0 (+ 0 ) | 1 (+ 1 ) | 1 (+ 0 ) |
| Kenya | 0 | 0 | 0 | 0 | 0 (+ 0 ) | 0 (+ 0 ) | 0 (+ 0 ) | 0 (+ 0 ) | 0 (+ 0 ) | 0 (+ 0 ) | 0 (+ 0 ) | 0 (+ 0 ) | 0 (+ 0 ) | 0 (+ 0 ) | 0 (+ 0 ) | 0 (+ 0 ) | 0 (+ 0 ) | 0 (+ 0 ) | 0 (+ 0 ) | 0 (+ 0 ) | 0 (+ 0 ) | 0 (+ 0 ) | 0 (+ 0 ) | 0 (+ 0 ) | 0 (+ 0 ) | 0 (+ 0 ) | 1 (+ 1 ) | 1 (+ 0 ) | 1 (+ 0 ) | 1 (+ 0 ) | 1 (+ 0 ) |
| Kuwait | 0 | 0 | 0 | 0 | 0 (+ 0 ) | 0 (+ 0 ) | 0 (+ 0 ) | 0 (+ 0 ) | 0 (+ 0 ) | 0 (+ 0 ) | 0 (+ 0 ) | 0 (+ 0 ) | 0 (+ 0 ) | 0 (+ 0 ) | 0 (+ 0 ) | 0 (+ 0 ) | 0 (+ 0 ) | 0 (+ 0 ) | 0 (+ 0 ) | 0 (+ 0 ) | 0 (+ 0 ) | 0 (+ 0 ) | 0 (+ 0 ) | 0 (+ 0 ) | 0 (+ 0 ) | 0 (+ 0 ) | 0 (+ 0 ) | 0 (+ 0 ) | 1 (+ 1 ) | 1 (+ 0 ) | 1 (+ 0 ) |
| Lebanon | 0 | 0 | 0 | 0 | 0 (+ 0 ) | 0 (+ 0 ) | 0 (+ 0 ) | 0 (+ 0 ) | 0 (+ 0 ) | 0 (+ 0 ) | 0 (+ 0 ) | 0 (+ 0 ) | 0 (+ 0 ) | 0 (+ 0 ) | 0 (+ 0 ) | 0 (+ 0 ) | 0 (+ 0 ) | 0 (+ 0 ) | 0 (+ 0 ) | 0 (+ 0 ) | 0 (+ 0 ) | 0 (+ 0 ) | 0 (+ 0 ) | 0 (+ 0 ) | 0 (+ 0 ) | 0 (+ 0 ) | 0 (+ 0 ) | 1 (+ 1 ) | 1 (+ 0 ) | 1 (+ 0 ) | 1 (+ 0 ) |
| Mongolia | 0 | 0 | 0 | 0 | 0 (+ 0 ) | 0 (+ 0 ) | 0 (+ 0 ) | 0 (+ 0 ) | 0 (+ 0 ) | 0 (+ 0 ) | 0 (+ 0 ) | 0 (+ 0 ) | 0 (+ 0 ) | 0 (+ 0 ) | 0 (+ 0 ) | 0 (+ 0 ) | 0 (+ 0 ) | 0 (+ 0 ) | 0 (+ 0 ) | 0 (+ 0 ) | 0 (+ 0 ) | 0 (+ 0 ) | 0 (+ 0 ) | 0 (+ 0 ) | 0 (+ 0 ) | 0 (+ 0 ) | 0 (+ 0 ) | 0 (+ 0 ) | 0 (+ 0 ) | 1 (+ 1 ) | 1 (+ 0 ) |
| Montenegro | 0 | 0 | 0 | 0 | 0 (+ 0 ) | 0 (+ 0 ) | 0 (+ 0 ) | 0 (+ 0 ) | 0 (+ 0 ) | 0 (+ 0 ) | 0 (+ 0 ) | 0 (+ 0 ) | 0 (+ 0 ) | 0 (+ 0 ) | 0 (+ 0 ) | 0 (+ 0 ) | 0 (+ 0 ) | 0 (+ 0 ) | 0 (+ 0 ) | 0 (+ 0 ) | 0 (+ 0 ) | 0 (+ 0 ) | 0 (+ 0 ) | 0 (+ 0 ) | 0 (+ 0 ) | 0 (+ 0 ) | 0 (+ 0 ) | 0 (+ 0 ) | 1 (+ 1 ) | 1 (+ 0 ) | 1 (+ 0 ) |
| Peru | 0 | 0 | 0 | 0 | 0 (+ 0 ) | 0 (+ 0 ) | 0 (+ 0 ) | 0 (+ 0 ) | 0 (+ 0 ) | 0 (+ 0 ) | 0 (+ 0 ) | 0 (+ 0 ) | 0 (+ 0 ) | 0 (+ 0 ) | 0 (+ 0 ) | 0 (+ 0 ) | 0 (+ 0 ) | 0 (+ 0 ) | 0 (+ 0 ) | 0 (+ 0 ) | 0 (+ 0 ) | 0 (+ 0 ) | 0 (+ 0 ) | 1 (+ 1 ) | 1 (+ 0 ) | 1 (+ 0 ) | 1 (+ 0 ) | 1 (+ 0 ) | 1 (+ 0 ) | 1 (+ 0 ) | 1 (+ 0 ) |
| Qatar | 0 | 0 | 0 | 0 | 0 (+ 0 ) | 0 (+ 0 ) | 0 (+ 0 ) | 0 (+ 0 ) | 0 (+ 0 ) | 0 (+ 0 ) | 0 (+ 0 ) | 0 (+ 0 ) | 0 (+ 0 ) | 0 (+ 0 ) | 0 (+ 0 ) | 0 (+ 0 ) | 0 (+ 0 ) | 0 (+ 0 ) | 0 (+ 0 ) | 0 (+ 0 ) | 0 (+ 0 ) | 0 (+ 0 ) | 0 (+ 0 ) | 0 (+ 0 ) | 0 (+ 0 ) | 0 (+ 0 ) | 0 (+ 0 ) | 1 (+ 1 ) | 1 (+ 0 ) | 1 (+ 0 ) | 1 (+ 0 ) |
| Saudi Arabia | 0 | 0 | 0 | 0 | 0 (+ 0 ) | 0 (+ 0 ) | 0 (+ 0 ) | 0 (+ 0 ) | 0 (+ 0 ) | 0 (+ 0 ) | 0 (+ 0 ) | 0 (+ 0 ) | 0 (+ 0 ) | 0 (+ 0 ) | 0 (+ 0 ) | 0 (+ 0 ) | 0 (+ 0 ) | 0 (+ 0 ) | 0 (+ 0 ) | 0 (+ 0 ) | 0 (+ 0 ) | 0 (+ 0 ) | 0 (+ 0 ) | 0 (+ 0 ) | 0 (+ 0 ) | 0 (+ 0 ) | 0 (+ 0 ) | 0 (+ 0 ) | 1 (+ 1 ) | 1 (+ 0 ) | 1 (+ 0 ) |
| Slovakia | 0 | 0 | 0 | 0 | 0 (+ 0 ) | 0 (+ 0 ) | 0 (+ 0 ) | 0 (+ 0 ) | 0 (+ 0 ) | 0 (+ 0 ) | 0 (+ 0 ) | 0 (+ 0 ) | 0 (+ 0 ) | 0 (+ 0 ) | 0 (+ 0 ) | 0 (+ 0 ) | 0 (+ 0 ) | 0 (+ 0 ) | 0 (+ 0 ) | 0 (+ 0 ) | 0 (+ 0 ) | 0 (+ 0 ) | 0 (+ 0 ) | 0 (+ 0 ) | 0 (+ 0 ) | 0 (+ 0 ) | 0 (+ 0 ) | 0 (+ 0 ) | 0 (+ 0 ) | 1 (+ 1 ) | 1 (+ 0 ) |
| Sri Lanka | 0 | 0 | 0 | 0 | 0 (+ 0 ) | 0 (+ 0 ) | 0 (+ 0 ) | 0 (+ 0 ) | 0 (+ 0 ) | 0 (+ 0 ) | 0 (+ 0 ) | 0 (+ 0 ) | 0 (+ 0 ) | 0 (+ 0 ) | 0 (+ 0 ) | 0 (+ 0 ) | 0 (+ 0 ) | 0 (+ 0 ) | 0 (+ 0 ) | 0 (+ 0 ) | 0 (+ 0 ) | 0 (+ 0 ) | 1 (+ 1 ) | 1 (+ 0 ) | 1 (+ 0 ) | 1 (+ 0 ) | 1 (+ 0 ) | 1 (+ 0 ) | 1 (+ 0 ) | 1 (+ 0 ) | 1 (+ 0 ) |
| Thailand | 0 | 0 | 0 | 0 | 0 (+ 0 ) | 0 (+ 0 ) | 0 (+ 0 ) | 0 (+ 0 ) | 0 (+ 0 ) | 0 (+ 0 ) | 0 (+ 0 ) | 0 (+ 0 ) | 0 (+ 0 ) | 0 (+ 0 ) | 0 (+ 0 ) | 0 (+ 0 ) | 0 (+ 0 ) | 0 (+ 0 ) | 0 (+ 0 ) | 0 (+ 0 ) | 0 (+ 0 ) | 0 (+ 0 ) | 0 (+ 0 ) | 0 (+ 0 ) | 0 (+ 0 ) | 0 (+ 0 ) | 0 (+ 0 ) | 0 (+ 0 ) | 0 (+ 0 ) | 1 (+ 1 ) | 1 (+ 0 ) |
| Europe | 0 | 0 | 0 | 0 | 0 (+ 0 ) | 0 (+ 0 ) | 0 (+ 0 ) | 1 (+ 1 ) | 3 (+ 2 , + 200 % ) | 3 (+ 0 ) | 8 (+ 5 , + 167 % ) | 10 (+ 2 , + 25 % ) | 17 (+ 7 , + 70 % ) | 20 (+ 3 , + 18 % ) | 23 (+ 3 , + 15 % ) | 33 (+ 10 , + 43 % ) | 43 (+ 10 , + 30 % ) | 56 (+ 13 , + 30 % ) | 66 (+ 10 , + 18 % ) | 78 (+ 12 , + 18 % ) | 95 (+ 17 , + 22 % ) | 117 (+ 22 , + 23 % ) | 132 (+ 15 , + 13 % ) | 159 (+ 27 , + 20 % ) | 181 (+ 22 , + 14 % ) | 218 (+ 37 , + 20 % ) | 259 (+ 41 , + 19 % ) | 311 (+ 52 , + 20 % ) | 401 (+ 90 , + 29 % ) | 501 (+ 100 , + 25 % ) | 507 (+ 6 , + 1 % ) |
| Asia | 0 | 0 | 0 | 0 | 0 (+ 0 ) | 0 (+ 0 ) | 0 (+ 0 ) | 0 (+ 0 ) | 0 (+ 0 ) | 0 (+ 0 ) | 0 (+ 0 ) | 0 (+ 0 ) | 0 (+ 0 ) | 1 (+ 1 ) | 1 (+ 0 ) | 1 (+ 0 ) | 1 (+ 0 ) | 2 (+ 1 , + 100 % ) | 2 (+ 0 ) | 5 (+ 3 , + 150 % ) | 8 (+ 3 , + 60 % ) | 14 (+ 6 , + 75 % ) | 27 (+ 13 , + 93 % ) | 42 (+ 15 , + 56 % ) | 61 (+ 19 , + 45 % ) | 84 (+ 23 , + 38 % ) | 117 (+ 33 , + 39 % ) | 173 (+ 56 , + 48 % ) | 246 (+ 73 , + 42 % ) | 349 (+ 103 , + 42 % ) | 361 (+ 12 , + 3 % ) |
| SouthAmerica | 0 | 0 | 0 | 0 | 0 (+ 0 ) | 0 (+ 0 ) | 0 (+ 0 ) | 0 (+ 0 ) | 0 (+ 0 ) | 0 (+ 0 ) | 0 (+ 0 ) | 0 (+ 0 ) | 0 (+ 0 ) | 0 (+ 0 ) | 1 (+ 1 ) | 1 (+ 0 ) | 1 (+ 0 ) | 1 (+ 0 ) | 3 (+ 2 , + 200 % ) | 4 (+ 1 , + 33 % ) | 5 (+ 1 , + 25 % ) | 8 (+ 3 , + 60 % ) | 13 (+ 5 , + 62 % ) | 18 (+ 5 , + 38 % ) | 23 (+ 5 , + 28 % ) | 32 (+ 9 , + 39 % ) | 39 (+ 7 , + 22 % ) | 45 (+ 6 , + 15 % ) | 67 (+ 22 , + 49 % ) | 98 (+ 31 , + 46 % ) | 101 (+ 3 , + 3 % ) |
| Africa | 0 | 0 | 0 | 0 | 0 (+ 0 ) | 0 (+ 0 ) | 0 (+ 0 ) | 0 (+ 0 ) | 0 (+ 0 ) | 0 (+ 0 ) | 0 (+ 0 ) | 0 (+ 0 ) | 0 (+ 0 ) | 0 (+ 0 ) | 0 (+ 0 ) | 0 (+ 0 ) | 0 (+ 0 ) | 0 (+ 0 ) | 0 (+ 0 ) | 0 (+ 0 ) | 0 (+ 0 ) | 0 (+ 0 ) | 0 (+ 0 ) | 0 (+ 0 ) | 0 (+ 0 ) | 0 (+ 0 ) | 4 (+ 4 ) | 4 (+ 0 ) | 7 (+ 3 , + 75 % ) | 13 (+ 6 , + 86 % ) | 13 (+ 0 ) |
| NorthAmerica | 0 | 0 | 0 | 0 | 1 (+ 1 ) | 1 (+ 0 ) | 1 (+ 0 ) | 4 (+ 3 , + 300 % ) | 6 (+ 2 , + 50 % ) | 6 (+ 0 ) | 8 (+ 2 , + 33 % ) | 8 (+ 0 ) | 10 (+ 2 , + 25 % ) | 12 (+ 2 , + 20 % ) | 13 (+ 1 , + 8 % ) | 16 (+ 3 , + 23 % ) | 21 (+ 5 , + 31 % ) | 26 (+ 5 , + 24 % ) | 36 (+ 10 , + 38 % ) | 45 (+ 9 , + 25 % ) | 50 (+ 5 , + 11 % ) | 65 (+ 15 , + 30 % ) | 77 (+ 12 , + 18 % ) | 93 (+ 16 , + 21 % ) | 110 (+ 17 , + 18 % ) | 132 (+ 22 , + 20 % ) | 163 (+ 31 , + 23 % ) | 196 (+ 33 , + 20 % ) | 248 (+ 52 , + 27 % ) | 300 (+ 52 , + 21 % ) | 301 (+ 1 , + 0 % ) |
| Oceania | 0 | 0 | 0 | 0 | 0 (+ 0 ) | 0 (+ 0 ) | 0 (+ 0 ) | 0 (+ 0 ) | 0 (+ 0 ) | 0 (+ 0 ) | 0 (+ 0 ) | 0 (+ 0 ) | 0 (+ 0 ) | 0 (+ 0 ) | 0 (+ 0 ) | 0 (+ 0 ) | 0 (+ 0 ) | 1 (+ 1 ) | 5 (+ 4 , + 400 % ) | 9 (+ 4 , + 80 % ) | 16 (+ 7 , + 78 % ) | 17 (+ 1 , + 6 % ) | 18 (+ 1 , + 6 % ) | 21 (+ 3 , + 17 % ) | 23 (+ 2 , + 10 % ) | 25 (+ 2 , + 9 % ) | 32 (+ 7 , + 28 % ) | 37 (+ 5 , + 16 % ) | 51 (+ 14 , + 38 % ) | 68 (+ 17 , + 33 % ) | 69 (+ 1 , + 1 % ) |
| Total p.a. Productivity | 0 | 0 | 0 | 0 | 1 ( NA % of prior year) | 0 ( NA % of prior year) | 0 ( NA % of prior year) | 4 ( NA % of prior year) | 4 ( 100 % of prior year) | 0 ( NA % of prior year) | 7 ( NA % of prior year) | 2 ( 29 % of prior year) | 9 ( 450 % of prior year) | 6 ( 67 % of prior year) | 5 ( 83 % of prior year) | 13 ( 260 % of prior year) | 15 ( 115 % of prior year) | 20 ( 133 % of prior year) | 26 ( 130 % of prior year) | 29 ( 112 % of prior year) | 36 ( 124 % of prior year) | 53 ( 147 % of prior year) | 51 ( 96 % of prior year) | 67 ( 131 % of prior year) | 69 ( 103 % of prior year) | 93 ( 135 % of prior year) | 124 ( 133 % of prior year) | 150 ( 121 % of prior year) | 242 ( 161 % of prior year) | 305 ( 126 % of prior year) | 27 ( 9 % of prior year) |
| Total Cummulative (CAVE: multi-country SRs counted once) | 0 | 0 | 0 | 0 | 1 (+ 1 ) | 1 (+ 0 ) | 1 (+ 0 ) | 5 (+ 4 , + 400 % ) | 9 (+ 4 , + 80 % ) | 9 (+ 0 ) | 16 (+ 7 , + 78 % ) | 18 (+ 2 , + 12 % ) | 27 (+ 9 , + 50 % ) | 33 (+ 6 , + 22 % ) | 38 (+ 5 , + 15 % ) | 51 (+ 13 , + 34 % ) | 66 (+ 15 , + 29 % ) | 86 (+ 20 , + 30 % ) | 112 (+ 26 , + 30 % ) | 141 (+ 29 , + 26 % ) | 177 (+ 36 , + 26 % ) | 230 (+ 53 , + 30 % ) | 281 (+ 51 , + 22 % ) | 348 (+ 67 , + 24 % ) | 417 (+ 69 , + 20 % ) | 510 (+ 93 , + 22 % ) | 634 (+ 124 , + 24 % ) | 784 (+ 150 , + 24 % ) | 1026 (+ 242 , + 31 % ) | 1331 (+ 305 , + 30 % ) | 1358 (+ 27 , + 2 % ) |

**Supplementary Table 2**: Validation of automated systematic review analysis function.

| **Item** | **FN** | **FP** | **TN** | **TP** | **sensitivity** | **specificity** | **precision** | **F1-score** | **accuracy** |
| --- | --- | --- | --- | --- | --- | --- | --- | --- | --- |
| Protocol | 6 | 3 | 50 | 12 | 0.666667 | 0.943396 | 0.8 | 0.727273 | 0.873239 |
| Research question | 0 | 9 | 0 | 62 | 1 | 0 | 0.873239 | 0.932331 | 0.873239 |
| Reporting of in/exclusion criteria | 0 | 3 | 0 | 68 | 1 | 0 | 0.957746 | 0.978417 | 0.957746 |
| Databases | 2 | 5 | 3 | 61 | 0.968254 | 0.375 | 0.924242 | 0.945736 | 0.901408 |
| Search data | 1 | 3 | 2 | 65 | 0.984848 | 0.4 | 0.955882 | 0.970149 | 0.943662 |
| Search string | 7 | 6 | 2 | 55 | 0.887097 | 0.25 | 0.901639 | 0.894309 | 0.814286 |
| Critical appraisal | 5 | 12 | 6 | 48 | 0.90566 | 0.333333 | 0.8 | 0.849558 | 0.760563 |
| Guidlines | 5 | 4 | 15 | 47 | 0.903846 | 0.789474 | 0.921569 | 0.912621 | 0.873239 |
| Study flowchart | 11 | 1 | 7 | 52 | 0.825397 | 0.875 | 0.981132 | 0.896552 | 0.830986 |
| Conflict of interest | 6 | 1 | 2 | 62 | 0.911765 | 0.666667 | 0.984127 | 0.946565 | 0.901408 |
| Involvement of at least 2 reviewers | 15 | 1 | 12 | 43 | 0.741379 | 0.923077 | 0.977273 | 0.843137 | 0.774648 |

*Abbreviations: FN, false negative; FP, false positive; TN, true negative; TP, true positive.*

**Supplementary Table 3**: Average systematic review quality score per country

| **Country** | **Mean quality** | **Median quality** | **Range min** | **Range max** | **Study count** |
| --- | --- | --- | --- | --- | --- |
| Argentina | 0.455 | 0.455 | 0.455 | 0.455 | 1 |
| Australia | 0.745 | 0.773 | 0.364 | 1 | 36 |
| Austria | 0.773 | 0.773 | 0.727 | 0.818 | 2 |
| Belgium | 0.75 | 0.773 | 0.455 | 1 | 8 |
| Brazil | 0.784 | 0.818 | 0.364 | 1 | 56 |
| Canada | 0.741 | 0.727 | 0.273 | 1 | 40 |
| Chile | 0.909 | 0.909 | 0.909 | 0.909 | 1 |
| China | 0.792 | 0.818 | 0.364 | 1 | 115 |
| Colombia | 0.742 | 0.818 | 0.455 | 1 | 6 |
| Croatia | 0.909 | 0.909 | 0.818 | 1 | 3 |
| Denmark | 0.737 | 0.727 | 0.364 | 1 | 19 |
| Egypt | 0.818 | 0.818 | 0.818 | 0.818 | 1 |
| France | 0.77 | 0.818 | 0.455 | 1 | 15 |
| Georgia | 0.636 | 0.636 | 0.545 | 0.727 | 2 |
| Germany | 0.727 | 0.727 | 0.364 | 1 | 33 |
| Greece | 0.727 | 0.773 | 0.455 | 0.909 | 4 |
| India | 0.826 | 0.909 | 0.455 | 1 | 11 |
| Indonesia | 0.788 | 0.818 | 0.636 | 0.909 | 3 |
| Iran | 0.803 | 0.818 | 0.364 | 1 | 48 |
| Ireland | 0.788 | 0.818 | 0.727 | 0.818 | 3 |
| Israel | 0.655 | 0.727 | 0.364 | 0.909 | 5 |
| Italy | 0.737 | 0.727 | 0.273 | 1 | 39 |
| Japan | 0.545 | 0.545 | 0.273 | 0.727 | 5 |
| Kenya | 0.909 | 0.909 | 0.909 | 0.909 | 1 |
| Kuwait | 0.636 | 0.636 | 0.636 | 0.636 | 1 |
| Lebanon | 0.909 | 0.909 | 0.909 | 0.909 | 1 |
| Malaysia | 0.828 | 0.818 | 0.545 | 1 | 9 |
| Mexico | 0.727 | 0.727 | 0.364 | 0.909 | 7 |
| Mongolia | 0.636 | 0.636 | 0.636 | 0.636 | 1 |
| Netherlands | 0.823 | 0.818 | 0.273 | 1 | 40 |
| New Zealand | 0.709 | 0.636 | 0.545 | 0.909 | 5 |
| Nigeria | 0.705 | 0.727 | 0.364 | 1 | 4 |
| Norway | 1 | 1 | 1 | 1 | 1 |
| Peru | 0.455 | 0.455 | 0.455 | 0.455 | 1 |
| Poland | 0.773 | 0.773 | 0.636 | 0.909 | 2 |
| Portugal | 0.727 | 0.818 | 0.364 | 0.909 | 6 |
| Russia | 0.795 | 0.773 | 0.636 | 1 | 4 |
| Singapore | 0.727 | 0.727 | 0.545 | 0.909 | 2 |
| South Africa | 0.909 | 0.909 | 0.909 | 0.909 | 2 |
| South Korea | 0.778 | 0.818 | 0.545 | 1 | 9 |
| Spain | 0.766 | 0.727 | 0.545 | 1 | 14 |
| Sri Lanka | 0.727 | 0.727 | 0.727 | 0.727 | 1 |
| Sweden | 0.807 | 0.864 | 0.545 | 1 | 8 |
| Switzerland | 0.81 | 0.818 | 0.545 | 1 | 11 |
| Taiwan | 0.682 | 0.682 | 0.364 | 1 | 2 |
| Thailand | 0.818 | 0.818 | 0.818 | 0.818 | 1 |
| Turkey | 0.848 | 0.909 | 0.727 | 0.909 | 3 |
| UK | 0.735 | 0.727 | 0.364 | 1 | 83 |
| USA | 0.681 | 0.727 | 0.182 | 1 | 107 |
| United Arab Emirates | 0.818 | 0.818 | 0.818 | 0.818 | 1 |
| Vietnam | 1 | 1 | 1 | 1 | 1 |

*Abbreviations: UK = United Kingdom, USA = United Stated of America.*
